# *Syzygium aromaticum* extract mediated, sustainable silver nanoparticle synergetic with heterocyclic antibiotic clarithromycin and their antimicrobial activities

**DOI:** 10.3389/fchem.2024.1513150

**Published:** 2025-01-15

**Authors:** Zehra Edis, Samir Haj Bloukh, Akram A. Ashames, Moawia M. Al-Tabakha, Moyad J. S. A. Shahwan, Hamed Abu Sara, Sai H. S. Boddu, Sohaib N. Khan, Ibrahim Haj Bloukh, Maram Eladdasy, Somayeh Sadeghi, Haneen Alkubaisi, Iman Haj Bloukh, Nageeb A. G. M. Hassan

**Affiliations:** ^1^ Department of Pharmaceutical Sciences, College of Pharmacy and Health Sciences, Ajman University, Ajman, United Arab Emirates; ^2^ Center of Medical and Bio-Allied Health Sciences Research, Ajman University, Ajman, United Arab Emirates; ^3^ Department of Clinical Sciences, College of Pharmacy and Health Sciences, Ajman University, Ajman, United Arab Emirates; ^4^ College of Dentistry, Ajman University, Ajman, United Arab Emirates

**Keywords:** silver nanoparticles, antibiotic resistance, clarithromycin, heterocyclic antibiotic, plant extract, clove

## Abstract

Microorganisms are becoming resistant to drugs and antimicrobials, making it a significantly critical global issue. Nosocomial infections are resulting in alarmingly increasing rates of morbidity and mortality. Plant derived compounds hold numerous antimicrobial properties, making them a very capable source to counteract resistant microbial strains. *Syzygium aromaticum* (Clove) extract has been proven by studies to contain active ingredients that demonstrate antibacterial, antifungal, antioxidant, and insecticidal properties. It has also been used historically for its pain relief especially for tooth ache. Clove extract derived nanoparticle synthesis is a promising method of combining therapeutics with metals at nanoscale. Such nanostructured systems in combination with the heterocyclic antibiotic clarithromycin could potentiate the action of plant extracts, decrease drug side effects and improve antimicrobial activity. In this study, clove extract (C) was successfully used to synthesize silver nanoparticles (AgNP) to create AgNPC and AgNPCA (A = clarithromycin). The two compounds underwent different analytical methods consisting of SEM, EDS, DLS, UV-vis, FTIR and XRD. These nanoparticles were used against a variety of 10 pathogens and exhibited very good to intermediate antibacterial properties. AgNPC resulted in better antibacterial properties and smaller nanoparticle size. This study demonstrates the potential of clove extract mediated AgNP synthesis in combination with and without the antibiotic clarithromycin.

## Graphical Abstract

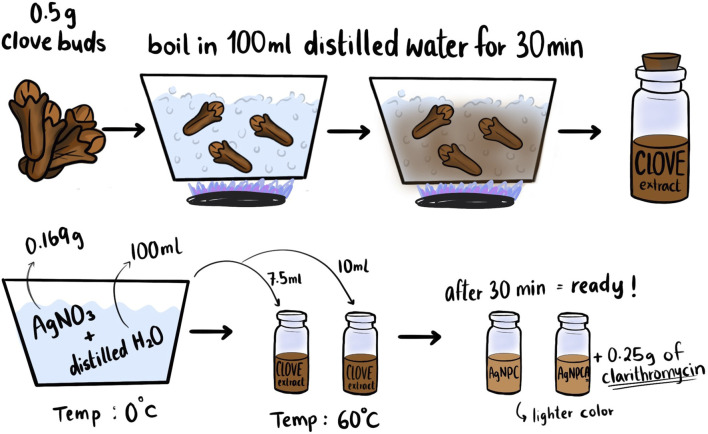

## 1 Introduction

Pathogenic microorganisms are a serious concern due to developing microbial resistance to antimicrobial agents (Baran et al., 2023; El-Sawy et al., 2024; Tang et al., 2023; Uddin et al., 2021). Pathogens learn to endure and survive what once were lethal concentrations, causing resistance to drugs, antibiotics and antimicrobials (Baran et al., 2023; El-Sawy et al., 2024; Tang et al., 2023; Uddin et al., 2021). This has become an alarming obstacle globally, causing a constant increase in the deaths from antimicrobial resistance (AMR) (Baran et al., 2023; El-Sawy et al., 2024; Tang et al., 2023; Uddin et al., 2021). Especially during COVID-19, AMR proved to be a major contributor to morbidity and mortality (Mahoney et al., 2021). Unfortunately, the uncontrolled use of antimicrobials to save COVID-19 patients in emergency wards and hospitals ameliorated AMR (Mahoney et al., 2021). Consequently, the search for new potent alternatives and synergistic agents to the commonly used antimicrobials and antibiotics has become an essential need for human survival (Uddin et al., 2021). Therefore, increasing numbers of investigations are dedicated to this pivotal pursuit, which include different approaches. Among the alternatives studied to combat antimicrobial resistant pathogens are plants with a rich spectrum of biocompounds (Adamczak et al., 2020; Ashraf et al., 2023; Barik et al., 2024; De Fazio et al., 2024; Di Lorenzo et al., 2021; Lalević al., 2023; Patanè et al., 2024). Many plants and essential oils have been used for medicine since ancient times due to their useful antimicrobial, antioxidative, anti-inflammatory and anticancer properties (Ashraf et al., 2023; Di Lorenzo et al., 2021; Edis et al., 2024). These medicinal plants integrate an abundance of bioactive compounds, which is an excellent defense that synergistically protects the plants against “opportunistic pathogens” and any other threats. Most medicinal plants and herbs are easy to find and low-cost, therefore convenient alternatives against AMR (Ashraf et al., 2023; Di Lorenzo et al., 2021; Edis and Bloukh, 2024; Edis et al., 2024; Edis et al., 2022; Edis and Bloukh, 2021; Edis and Bloukh, 2020; Hameed et al., 2021; Lakhan et al., 2020; Maggini et al., 2024; Mohammed et al., 2021; Murtaza et al., 2024; Ricardo-Rodrigues et al., 2024; Romanescu et al., 2023; Singh et al., 2024; Xu et al., 2023). Medicinal plants can be applied on wounds, as well as dental and oral care (Ashraf et al., 2023; Di Lorenzo et al., 2021; Edis and Bloukh, 2024; Edis et al., 2024; Edis et al., 2022; Edis and Bloukh, 2021; Edis and Bloukh, 2020; Hameed et al., 2021; Lakhan et al., 2020; Maggini et al., 2024; Mohammed et al., 2021; Murtaza et al., 2024; Ricardo-Rodrigues et al., 2024; Romanescu et al., 2023; Singh et al., 2024; Xu et al., 2023).

Clove (*Syzygium aromaticum*) is a commonly known plant belonging to the Myrtaceae family (Singh et al., 2024). Clove possesses effective antimicrobial properties and is a frequently used preservative (Hameed et al., 2021; Lakhan et al., 2020; Maggini et al., 2024; Mohammed et al., 2021; Murtaza et al., 2024; Ricardo-Rodrigues et al., 2024; Singh et al., 2024; Xu et al., 2023). The clove plant’s flower buds contain the greatest essential oil concentration in the plant (Hameed et al., 2021; Maggini et al., 2024; Ricardo-Rodrigues et al., 2024; Singh et al., 2024; Xu et al., 2023). Clove attracted attention due to its antioxidant, antimicrobial, antinociceptive, antiviral, and cytotoxic properties (Hameed et al., 2021; Singh et al., 2024; Xu et al., 2023). It represents one of the main plant sources of phenolic compounds such as flavonoids, hydroxybenzoic acids, hydroxycinnamic acids and hydroxyphenyl propenes (Singh et al., 2024). Clove is one of the most valuable sources of phenolic compounds, such as quercetin and kaempferol, ellagic acid, caffeic acid, as well as ferulic acid (Singh et al., 2024). Eugenol is the main compound found in clove (Hameed et al., 2021; Lakhan et al., 2020; Singh et al., 2024). It has been used in dental applications for pain relieve and for its anti-inflammatory properties (Hameed et al., 2021; Maggini et al., 2024; Singh et al., 2024). The topical use of clove oil is a known and ancient remedy for the relief of tooth pain, which patients still utilize even in modern times (Hameed et al., 2021; Maggini et al., 2024; Singh et al., 2024). Clove is increasingly used for the biosynthesis of nanoparticles and offers interesting results (Hameed et al., 2021; Lakhan et al., 2020; Mohammed et al., 2021; Murtaza et al., 2024; Singh et al., 2024; Xu et al., 2023). Recently, nanoparticles and plant extracts are used as anti-cancer agents within different applications (Chen et al., 2023; Cheng et al., 2023; Lou et al., 2021; Shi et al., 2022; Tang et al., 2017; Wang et al., 2025; Wang et al., 2024; Zeng et al., 2020).

Another option against AMR is the utilization of nanotechnology with silver nanoparticles (Al Aboody, 2019; Al-Otibi et al., 2021; Ankegowda et al., 2020; Barik et al., 2024; Bloukh et al., 2020; Bruna et al., 2021; Corciovă et al., 2024; Desai et al., 2023; El-Kahky et al., 2021; Haj Bloukh et al., 2021; Hernández-Venegas et al., 2023; Mateo and Jiménez, 2022; Menichetti et al., 2023; Mussin and Giusiano, 2024; Nguyen et al., 2023; Pereira et al., 2024; Reda et al., 2019; Riau et al., 2019; Samuggam et al., 2021; Sukhanova et al., 2018; Suvandee et al., 2022). Silver (Ag) has been studied throughout history for its exceptionally potent bactericidal and antimicrobial properties, which can be used against AMR (Bruna et al., 2021; Desai et al., 2023; Haj Bloukh et al., 2021; Hernández-Venegas et al., 2023; Mateo and Jiménez, 2022; Nguyen et al., 2023; Pereira et al., 2024; Riau et al., 2019). Ag is generally unreactive, but ionizes due to the presence of oxygen and moisture in the tissues (Desai et al., 2023; Hernández-Venegas et al., 2023; Pereira et al., 2024; Riau et al., 2019; Singh et al., 2024). This ionization results in the release of silver cations (Ag^+^), which are biologically active silver ions (Al Aboody, 2019; Desai et al., 2023; Haj Bloukh et al., 2021; Hernández-Venegas et al., 2023; Menichetti et al., 2023; Pereira et al., 2024; Riau et al., 2019; Singh et al., 2024). Ag^+^-ions then attach themselves to thiol groups, anionic ligands of proteins and then to the bacterial cell membrane (Al Aboody, 2019; Desai et al., 2023; Haj Bloukh et al., 2021; Hernández-Venegas et al., 2023; Menichetti et al., 2023; Pereira et al., 2024; Riau et al., 2019; Singh et al., 2024). Once it binds to the cell membrane, it will induce pinocytosis which is the penetration of the bacterial cell wall, leading to denaturation of proteins and the growth arrest of bacteria by enzymes (Al Aboody, 2019; Desai et al., 2023; Haj Bloukh et al., 2021; Hernández-Venegas et al., 2023; Menichetti et al., 2023; Pereira et al., 2024; Riau et al., 2019; Singh et al., 2024).

Currently, the use of silver nanoparticles (AgNP) can be found in water treatments, wound care products, antiseptic sprays, medical devices and cosmetics, for the protection against pathogens (Al Aboody, 2019; Al-Otibi et al., 2021; Ankegowda et al., 2020; Barik et al., 2024; Bloukh et al., 2020; Bruna et al., 2021; Corciovă et al., 2024; Desai et al., 2023; El-Kahky et al., 2021; Haj Bloukh et al., 2021; Hernández-Venegas et al., 2023; Mateo and Jiménez, 2022; Menichetti et al., 2023; Mussin and Giusiano, 2024; Nguyen et al., 2023; Pereira et al., 2024; Reda et al., 2019; Riau et al., 2019; Samuggam et al., 2021; Sukhanova et al., 2018; Suvandee et al., 2022). Silver nanoparticles and plants can be used synergistically due to their outstanding individual properties, giving us greater benefits and an excellent chance to win the battle against AMR (Al Aboody, 2019; Desai et al., 2023; Haj Bloukh et al., 2021; Hernández-Venegas et al., 2023; Menichetti et al., 2023; Pereira et al., 2024; Riau et al., 2019; Singh et al., 2024).

Medicinal plants and nanoparticles can be used in combination with antibiotics to achieve a synergistic effect against resistant pathogens (Khan et al., 2022). Clarithromycin is a commonly used semisynthetic, heterocyclic macrolide antibiotic (Domanovich-Asor et al., 2021; Khan et al., 2022; Lebel, 1993; Takemori et al., 2020; Yamamoto et al., 2021). It is a part of the 14-membered macrolide antibiotic family along with erythromycin and roxithromycin (Domanovich-Asor et al., 2021; Khan et al., 2022; Lebel, 1993; Takemori et al., 2020; Yamamoto et al., 2021). Clarithromycin is an acid-stable equivalent of erythromycin having a methoxy substitution at C-6 of the erythronolide ring (Domanovich-Asor et al., 2021; Khan et al., 2022; Lebel, 1993; Takemori et al., 2020; Yamamoto et al., 2021). This structural difference inhibits the conversion to inactive spiroketal forms in the stomach and enhances the bioavailability and GI tolerance after taking a dose orally. This difference will increase its bactericidal activity when compared to erythromycin (Khan et al., 2022; Lebel, 1993; Takemori et al., 2020; Yamamoto et al., 2021). The antibacterial activity is correlated with its ability to inhibit protein synthesis in bacteria, which is achieved by the binding of the molecules to the subunit 50S of the bacterial ribosome (Khan et al., 2022; Lebel, 1993; Takemori et al., 2020; Yamamoto et al., 2021). The metabolism of clarithromycin in humans produces 14-hydroxy clarithromycin, which promotes an additive or synergistic action to the action of the parent compound against chosen pathogens (Khan et al., 2022; Lebel, 1993; Takemori et al., 2020; Yamamoto et al., 2021). Clarithromycin has a rapid first-pass effect in the liver following its intestinal absorption. Due to its acid stability property, it has a half-life of 5–7 h when taken orally with the dose of 500 mg, meaning it needs to be administered every 12 h (Khan et al., 2022; Lebel, 1993; Takemori et al., 2020; Yamamoto et al., 2021). Increasing antimicrobial resistance against clarithromycin in comparison to other common antibiotics is alarming. Synergetic mixtures of AgNP and antibiotics in colloidal solutions are often governed by complex processes (Domanovich-Asor et al., 2021; Pani and Chandrasekaran, 2024; Ullah et al., 2023). Recently published studies combine AgNPs with antibiotics, including clarithromycin (Adil et al., 2023; Dove et al., 2023; Hasoon et al., 2024; Ormeño-Martínez et al., 2024; Samari-Kermani et al., 2021; Yang et al., 2017; Zúñiga-Miranda et al., 2023; Ruban et al., 2023). The target is to lower the dosages to mitigate AgNP toxicity and development of resistance towards these new compounds (Adil et al., 2023; Dove et al., 2023; Hasoon et al., 2024).

In this study, we used a clove bud extract (C) mediated silver nanoparticle (AgNP) synthesis in form of AgNPC. We also aimed at introducing clarithromycin into the formulation to study the changes within the compound. The antimicrobial properties of the two title compounds AgNPC and AgNPCA against a panel of 10 microorganisms were investigated. The study revealed higher antimicrobial activities and smaller nanoparticle size for AgNPC compared to AgNPCA. The reasons for these results were found in the molecular changes, when clarithromycin is present. Amalgamation of AgNPC with clarithromycin increases the size of the nanoparticles, causes aggregation (Adil et al., 2023; Dove et al., 2023; Hasoon et al., 2024; Ruban et al., 2023). A comparison between SEM and DLS sourced particle size measurements showed a slight difference and pointed out a possible organic layer around AgNPCA caused by the availability of clarithromycin (Tarrés et al., 2022). However, alleviated particle size in AgNPCA along with agglomeration did not counteract the antimicrobial properties. AgNPCA, the synergistic compound between clove-bud mediated AgNP and clarithromycin, inhibited pathogens, which are resistant to pure clarithromycin. Bacterial strains were in general more susceptible to AgNPC, followed by AgNPCA and lastly to clarithromycin alone. The results indicate possible applications for the title compounds in wound treatment. Further investigations like *in vitro* studies and cytotoxicity analysis are needed to confirm the uses in the medical field.

## 2 Materials and methods

### 2.1 Materials

Dry clove buds were purchased from the local market of UAE. Sterile filter paper Whatman 150 mm were purchased from GE Healthcare (Amersham Place Little Chalfont, Buckinghamshire, HP7 9NA, United States). Sodium hydroxide (NaOH) pellets, chlarithromycin and silver nitrate (AgNO_3_) were provided from Sigma-Aldrich Chemical Co. (St. Louis, MO, United States). The same company supplied the reference strains *E. coli* WDCM 00013 Vitroids, *K. pneumoniae* WDCM 00097 Vitroids, *P. aeruginosa* WDCM 00026 Vitroids, *B. subtilis* WDCM 0003 Vitroids, and *C. albicans* WDCM 00054 Vitroids. Mueller Hinton Broth (MHB), Sabouraud Dextrose broth and ethanol were also procured from Sigma Aldrich. Further strains consisting of *P. mirabilis* ATCC 29906, *S. aureus* ATCC 25923, *S. pyogenes* ATCC 19615, *E. faecalis* ATCC 29212, and *S. pneumoniae* ATCC 49619 were purchased from Liofilchem (Roseto degli Abruzzi, TE, Italy). Himedia (Jaitala Nagpur, Maharashtra, India) provided sterile filter paper discs with a diameter of 6 mm. Liofilchem Diagnostici (Roseto degli Abruzzi (TE), Italy) supplied antibiotic discs of nystatin (9078, 100 IU/disc) and gentamicin (9125, 30 µg/disc), as well as disposable sterilized Petri dishes containing Mueller Hinton II agar and McFarland standard sets. All utilized reagents were of analytical grade. All experiments were done under sterile conditions with ultrapure water and absolute ethanol.

### 2.2 Preparation of clove (C) extract

The clove buds were finely grinded. 0.5 g of the obtained clove powder was filled into a 250 mL beaker with 100 mL of distilled water, covered and heated to 60°C with stirring for 30 min. The resulting light brown extract was cooled down to room temperature and filtered by a Whatman 150 mm filter paper into a 250 mL flask. 30 mL and 40 mL of clove extract were diluted with 70 mL and 60 mL ultra-distilled water, respectively. The prepared stock solution was transferred into brown, screw capped bottles and stored at 3°C in the fridge until further usage equations should be inserted in editable format from the equation editor.

### 2.3 Preparation of AgNPC and AgNPCA

AgNO_3_ solution was prepared by adding 0.169 g of AgNO_3_ into 100 mL of distilled water at 0°C and 10 min of constant stirring. After that, 7.5 mL and 10 mL of these AgNO_3_ solutions were added into 100 mL of the prepared 30% and 40% clove stock solutions, respectively. The silver nitrate solution was added during constant stirring into the clove extract stock solution at a temperature of 60°C. After 30 min continuous stirring, color changes were observed in the solution from dark brown to light brown. Few drops (1-2 drops) of HCl at 0°C and 10 min of stirring were added into AgNPC to adjust the pH from 8.5 to 8.3. AgNPCA is prepared by first dissolving 0.125 g clarithromycin into 250 mL of distilled water. Then, 25 mL of this clarithromycin solution was added under constant stirring into a 40%-AgNPC solution at 0°C within 10 min.

### 2.4 Characterization of AgNPC and AgNPCA

Morphology and composition of AgNPC and AgNPCA were studied by SEM/EDS, UV-vis, FTIR, and x-ray diffraction (XRD).

#### 2.4.1 Scanning electron microscopy (SEM) and energy-dispersive X-ray spectroscopy (EDS)

The SEM (scanning electron microscopy) and EDS (energy-dispersive X-ray spectroscopy) analysis was performed with the Thermofisher scientific APREO 2C SEM (Waltham, Massachusetts, United States 02451). The analysis was conducted at 10 kV after being diluted with distilled water, dropped onto a carbon-coated copper tape, dried and covered with a gold coating with the Quorum Technology Mini Sputter Coater.

#### 2.4.2 Size and zeta potential analysis

Calculating the average size, size distribution, zeta potential, as well as polydispersity index (PDI) of AgNPC and AgNPCA was achieved by Dynamic light scattering (DLS) analysis by a Horiba SZ-100 (Palaiseau, France).

#### 2.4.3 UV-vis spectrophotometry (UV-Vis)

The UV-vis analysis of AgNPC and AgNPCA was done on a Shimadzu spectrophotometer model 2600i (Kyoto, Japan). Measurements included the wavelength spectrum from 195 to 800 nm.

#### 2.4.4 Fourier-transform infrared spectroscopy (FTIR)

AgNPC and AgNPCA underwent Fourier Transform Infrared (FTIR) analysis within the spectral range of 400–4,000 cm^−1^ by utilizing a Shimadzu Attenuated Total Reflectance (ATR) IR spectrometer equipped with a Diamond window (Kyoto, Japan).

#### 2.4.5 X-ray diffraction (XRD)

The X-ray diffraction analysis was performed by using a BRUKER D8 Advance (Karlsruhe, Germany). The study used a Two Theta configuration, with a time per step of 0.5 s and a step size of 0.03 equipped with Cu radiation at a wavelength of 1.54060 Å.

### 2.5 Antimicrobial studies

The title compounds AgNPC and AgNPCA were tested against the ten reference strains, which included *S. aureus* ATCC 25923, *S. pneumoniae* ATCC 49619, *E. faecalis* ATCC 29212, *S. pyogenes* ATCC 19615, *B. subtilis* WDCM 0003 Vitroids, *K. pneumoniae* WDCM 00097 Vitroids, *E. coli* WDCM 00013 Vitroids, *P. aeruginosa* WDCM 00026 Vitroids, *P. mirabilis* ATCC 29906 and *C. albicans* WDCM 00054 Vitroids. Nystatin and gentamicin and nystatin were utilized as positive controls. Negative controls included pure ethanol and ultrapure water. These negative controls showed no inhibition zone. Every test was done thrice, and the average results were reported.

#### 2.5.1 Bacterial strains and culturing

AgNPC and AgNPCA were tested against ten reference, standard microbial strains consisting of *S. aureus* ATCC 25923, *S. pneumoniae* ATCC 49619, *E. faecalis* ATCC 29212, *S. pyogenes* ATCC 19615, *B. subtilis* WDCM 0003 Vitroids, *K. pneumoniae* WDCM 00097 Vitroids, *E. coli* WDCM 00013 Vitroids, *P. aeruginosa* WDCM 00026 Vitroids, *P. mirabilis* ATCC 29906 and *C. albicans* WDCM 00054 Vitroids. The reference strains were stored at −20°C and then revived by inoculating fresh microbes into Mueller Hinton Broth (MHB). These prepared strains were then kept at 4°C until further use.

#### 2.5.2 Procedure for zone of inhibition (ZOI) plate studies

The zone of inhibition (ZOI) plate method was used to investigate the antimicrobial activities of AgNPC and AgNPCA against the 10 microbial reference strains (Bauer et al., 1959). All the nine bacterial reference strains were suspended in 10 mL of Mueller-Hinton broth (MHB) and then incubated for 2–4 h at 37°C. Only the fungus *C. albicans* WDCM 00054 was cultured in Sabouraud Dextrose broth at 30°C. The microbial cultures were adjusted to 0.5 McFarland standard and 100 μL of microbial culture was evenly seeded with sterile cotton swaps on ready-made disposable, sterilized Petri dishes. These plates were dried for 10 min at ambient conditions and then utilized for the antimicrobial testing.

#### 2.5.3 Disc diffusion method (DD)

The antimicrobial testing of AgNPC and AgNPCA followed the guidelines of the Clinical and Laboratory Standards Institute (CLSI) (CLSI, 2019). Sterile filter paper discs were soaked in 2 mL AgNPC and AgNPCA solutions of various concentrations for 24 h at ambient conditions. Afterwards, the discs were dried at ambient conditions for 24 h. Nystatin and gentamycin antibiotic discs were utilized as positive controls. The clear area around the soaked disk is measured by a ruler to the nearest millimeter and is the diameter of zone of inhibition (ZOI). No inhibition zone around the disk is considered as resistant (R) against the reference strain.

### 2.6 Statistical analysis

The statistical analysis was done utilizing SPSS software (version 17.0, SPSS Inc., Chicago, IL, United States), with data presented in mean values. The significance between groups was determined through one-way ANOVA. Statistical significance value was defined as p < 0.05.

## 3 Results and discussion

AMR is a dangerous threat to human health the future of its existence (Baran et al., 2023; Tang et al., 2023; Uddin et al., 2021). Multi-drug resistant pathogens cause higher morbidity and mortality rates among immunocompromised patients worldwide (Baran et al., 2023; Tang et al., 2023; Uddin et al., 2021). Alternative agents are needed to support or replace antimicrobials. Following this trend, we present in this study biosynthesized silver nanoparticles AgNPC and AgNPCA and their antimicrobial properties. The two title compounds were characterized by diverse analytical methods, as well as tested against a selection of 10 reference microbial strains.

### 3.1 Electron microscope (SEM) and energy-dispersive X-ray spectroscopic (EDS) analysis

**FIGURE 1:**
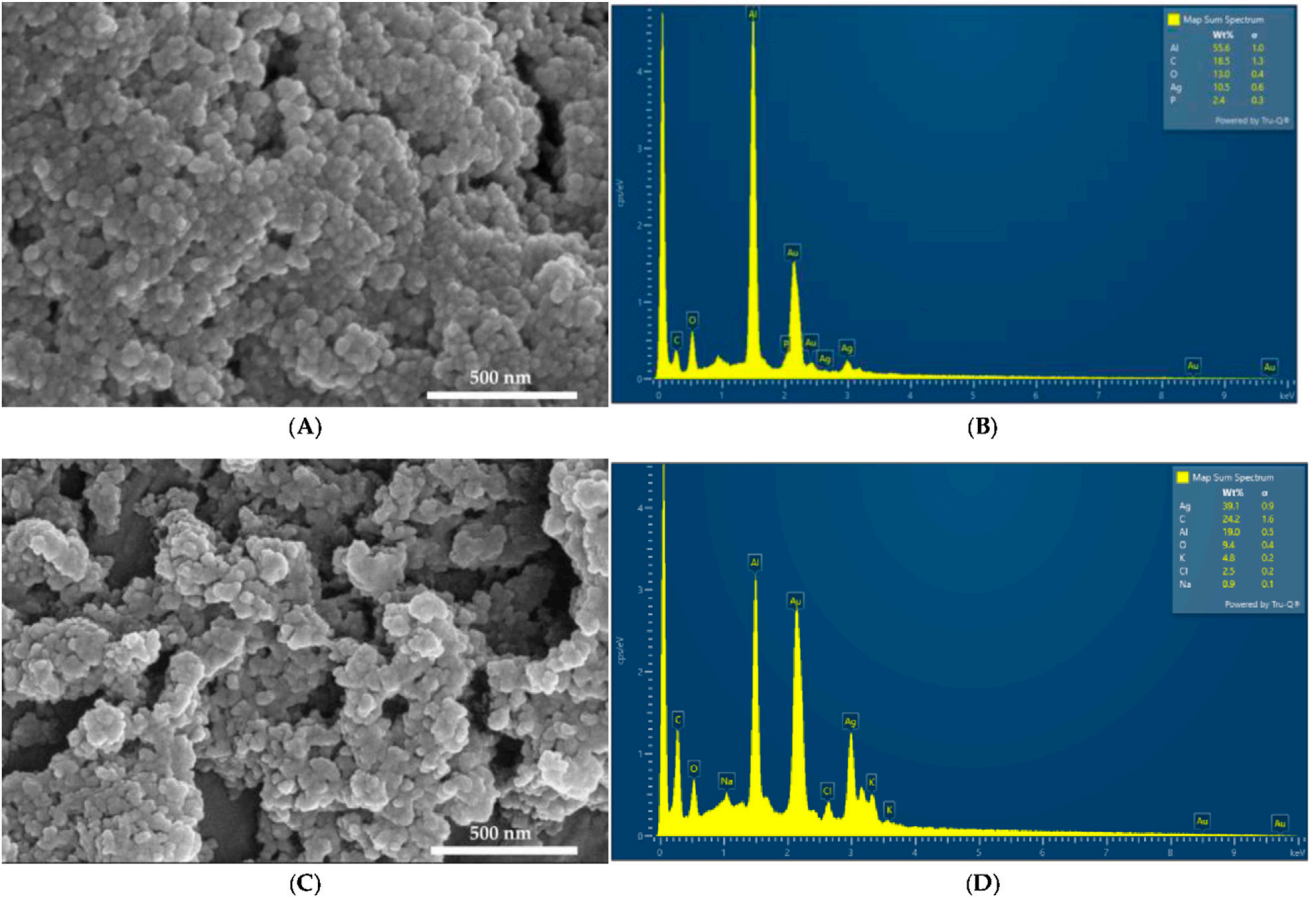
SEM and EDS analysis. AgNPC **(A)** SEM; **(B)** EDS; AgNPCA **(C)** SEM; **(D)** EDS.

The morphology and composition of AgNPCA and AgNPC were studied by SEM and EDS analysis, respectively (Figure 1).

Figure 1A reveals a smooth surface texture morphology of AgNPC with small, spherical structures. In contrast, Figure 1C looks a bit more heterogeneous with slightly bigger, flatter entities in AgNPCA (Figure 1A, Supplementary Material S1–S4). These changes in size and morphology confirm the incorporation of clarithromycin into AgNPCA. The EDS displays in Figure 1B the presence of Al (55.6%), C (18.5%), O (13.0%), Ag (10.5%) and P (2.4%). Figure 1D presents Ag (39.1%), C (24.2%), Al (19.0%), O (9.4%), K (4.8%), Cl (2.5%) and Na (0.9%). In both samples, aluminum and gold are found due to the sample holder and the coating process, respectively. Both samples show high purity by the EDS analysis. Oxygen and carbon are available in both samples due to the abundance of biomolecules originating from the clove extract. The peak of Ag appears in both EDS between 3 and 4 keV in agreement with previous reports (Bloukh et al., 2020; Haj Bloukh et al., 2021; Mussin and Giusiano, 2024; Singh et al., 2024). Figure 1D shows a small peak around 0.5 keV for AgNPCA, which indicates also the existence of Ag_2_O through oxidation (Singh et al., 2024). K and Na, as well as Cl are introduced through clarithromycin in form of inactive ingredients within the antibiotic (Pani and Chandrasekaran, 2024). The addition of few drops of HCl to stabilize the pH, the nanoparticles and the clarithromycin contributed also to the detection of Cl (Ullah et al., 2023). The particle size measurements by SEM on both samples provided 7.3 ± 0.3 nm for AgNPC and 7.7 ± 0.6 nm for AgNPCA (Supplementary Material S4).

### 3.2 Dynamic light scattering (DLS) and zeta potential analysis

The DLS and Zeta Potential analysis provide crucial information regarding the size, agglomeration, distribution, and stability of nanoparticles in solution. Table 1 shows the results of the DLS measurements of AgNPC and AgNPCA.

**TABLE 1:** DLS and Zeta Potential results for AgNPCA and AgNPC.

Sample	Zeta potential (mV)	Particle size mean (nm)	Z-Average (nm)	Polydispersity index (PDI)
AgNPC	−34.9	12.8 ± 11.2	65.6	0.458
AgNPCA	−11.1	36.0 ± 34.3	-	-

The DLS results of the AgNPCA and AgNPC reveal an average size of 36.0 nm and 12.8 nm, respectively (Table 1). Further peak intensities are not available in the spectrum. Clarithromycin and AgNPC attract each other because of their opposite charges leading to aggregation and increase in particle size (Table 1).

AgNPC has a polydispersity index (PDI) of 0.458 additionally to the average size of 12.8 nm. This data verifies a polydisperse sample, in which the phenolic compounds in the clove extract effectively functioned as capping and reducing agents (Desai et al., 2023; Haj Bloukh et al., 2021). The PDI and Z-average for AgNPCA could not be calculated due to the increase in particle size, instability and agglomeration. Our DLS has a limit up to 50, any measurement above that will not be reported. Therefore, a PDI value increase above 50 is expected for AgNPCA. This increase in PDI from AgNPC to AgNPCA verifies, that the nanocompound is strongly polydisperse and clarithromycin contributed to their synthesis (Adil et al., 2023).

Hence, zeta (ζ) potential analysis allows predictions about AgNP stability within a colloidal suspension (Desai et al., 2023; Haj Bloukh et al., 2021). The zeta (ζ) potential of AgNPC and AgNPCA are −34.9 and −11.1 mV, respectively (Table 1). Their negative values point towards negatively charged AgNP surfaces (Bloukh et al., 2020; Desai et al., 2023; Haj Bloukh et al., 2021; Sukhanova et al., 2018). The negative charge of −34.9 mV indicates that AgNPC solution is stable, while AgNPCA appears to form agglomerates due to its much bigger zeta potential of −11.1 mV. The negative charge of −34.9 mV indicates that AgNPC solution is stable, while AgNPCA appears to form agglomerates due to its much bigger zeta potential of −11.1 mV. However, apart from electrostatic stabilization, steric stabilization from clove biocompounds and clarithromycin molecules surrounding the nanoparticles could sustain stability, although the zeta potential approaches towards zero (Adil et al., 2023; Bloukh et al., 2020; Desai et al., 2023; Haj Bloukh et al., 2021; Pani and Chandrasekaran, 2024; Sukhanova et al., 2018). Possibly, higher cytotoxicity is expected in AgNPCA compared to AgNPC, because NP with ζ higher than ±30 mV are more stable and do not agglomerate in general (Desai et al., 2023; Sukhanova et al., 2018). The DLS results confirm a slight decrease in colloidal stability for AgNPCA, while steric stabilization by surrounding clarithromycin and clove biocompunds is achieved (Adil et al., 2023).

However, in comparison to Pani et al., clarithromycin addition into a suspension with polymers resulted in changes of the zeta-potential, size and PDI (Pani and Chandrasekaran, 2024). Clarithromycin is a large molecule with several electronegative oxygen atoms, nitrogen atoms and hydroxide groups in the periphery of the macrocyclic molecule. Once it is introduced into AgNPC, the zeta potential changes to a higher, but still negative number (−11.1 mV) compared to AgNPC with −34.9 mV. This change is also seen in the study of Pani et al., when clarithromycin was added into the nanoparticle suspension (Pani and Chandrasekaran, 2024). The zeta potential changed from −52.2 mV to −14.3 mV with clarithromycin (Pani and Chandrasekaran, 2024). Pereira et al. added chitosan to AgNP and observed similar increase in the zeta potential from −26.3 mV with the chitosan coating towards −15.9 mV (Pereira et al., 2024). The authors stated, that chitosan introduced a slight positive charge, increasing the zeta potential slightly (Pereira et al., 2024). Our results also confirm a slight decrease of negative charge towards higher zeta potential by adding clarithromycin (Table 1). Therefore, the zeta potential changes confirm changes on the AgNP surface and size of the resulting AgNPCA. In this regard, Pani et al. have seen a change in AgNP size from initially 545.7–1,289.8 nm, while our results show an increase from 12.8 towards 36 nm, when clarithromycin was introduced (Pani and Chandrasekaran, 2024). The reported PDI for Pani et al. was 0.286 initially and changed to 0.056 after adding clarithromycin (Pani and Chandrasekaran, 2024). In comparison, our DLS analysis shows a PDI of 0.458, while AgNPCA was not detected (Table 1). All the DLS results point towards aggregation induced by an increasing layer of organic molecules around the silver nanoparticles after adding clarithromycin into the formulation. This assumption is confirmed by a comparison between the particle size measurements of SEM and DLS analysis (Table 1, Supplementary Material S4). The SEM measurements provide 7.3 ± 0.3 nm for AgNPC and 7.7 ± 0.6 nm for AgNPCA, while Table 1 mentions hydrodynamic diameters of 12.8 and 36 nm, respectively (Supplementary Material S4). Accordingly, the difference between SEM and DLS analysis points towards 28.3 nm around AgNPCA and 5.5 nm around AgNPC. These measurements highlight the thickness of the organic layer around the nanoparticles.

In conclusion, AgNPC presents as a stable, homogenous bio-nano-compound, with small average particle size and high stability. The clove-based phenolic compounds with their -OH and -C=O groups surrounded the AgNP surface and prevented agglomeration through further secondary nucleation (Bloukh et al., 2020; Desai et al., 2023; Haj Bloukh et al., 2021, 2023; Singh et al., 2024). This monolayer of plant phenolic compounds is compromised by the addition of clarithromycin. The antibiotic possibly removes the layer of phenolic compounds and exposes AgNP to agglomeration, although steric stabilization is achieved, an increased size and changes in the antimicrobial activities are observed (Bloukh et al., 2020; Desai et al., 2023; Haj Bloukh et al., 2021, 2023; Singh et al., 2024).

### 3.3 X-ray diffraction (XRD) of AgNPC and AgNPCA

**FIGURE 2:**
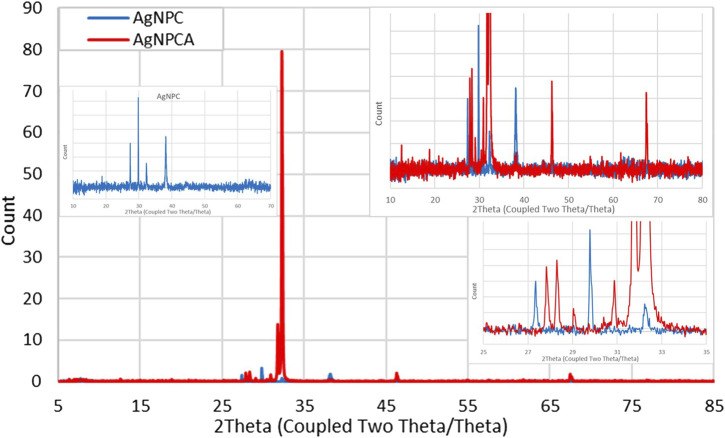
X-ray diffraction (XRD) study of AgNPCA (red) and AgNPC (blue).

The XRD analysis of AgNPC and AgNPCA depict the composition and the crystalline nature of the two samples (Figure 2).

The XRD analysis reveals in both nano-compounds crystalline phases of pure AgNP (Figure 2). AgNPCA (red) shows sharp, very strong peaks with 2θ values around 32° while AgNPC (blue) has almost similar, but much weaker reflections with 2Theta values of 18.80° (001), 27.35° (001), 29.78° (003), 32.24° (001), 38.13° (111), 46.33° (200) and 62.61° (003) for AgNP (Figure 2; Table 2) (Al Aboody, 2019; Ankegowda et al., 2020; El-Kahky et al., 2021; Al-Otibi et al., 2021; Haj Bloukh et al., 2021; Samuggam et al., 2021; Suvandee et al., 2022).

**TABLE 2:** XRD study of AgNPCA, AgNPC, clarithromycin (Antibiotic) and other investigations with their calculated planes (2Theta^o^).

	AgNPCA	AgNPC	1	2	3	4	5	6	7	Planes
AgNP	18.08 w 28.30 m 30.88 m 31.73 m	18.80 w 27.35 vw 29.78 w 32.24 vw	19.02 28.35 30.89 32.34	- - - -	- - - -	- - - 32	- - - -	- - - -	- - - -	(001) (001) (003) (001)
AgNP (JCPDS 04-0783)	38.28 w 46.24 m 67.44 m 76.89 vw	38.13 w 46.33 vw 62.61 vw 75.99 vw	38.08 - - -	38.4 44.5 64.8 77.4	38.61 46.43 65.52 78.28	- 47 68 78	38.25 44.43 64.67 77.59	38.08 44.21 64.42 77.32	- - - -	(111) (200) (220) (311)
AgCl	29.04 w 30.40 w	- -	- -	- -	- -	- -	- -	- -	- -	
Ag_2_O (JCPDS 041-1104)	27.83 m 32.24 vs 37.90 vw 54.89 vw 61.72 vw 72.9 vw	- - - - - -	- - - - - -	26.7 32.7 37.9 54.9 65.5 69.0	- - - - - -	- - - - - -	28 32 46 - - -	- - - - - -	- - - - - -	(110) (111) (200) (220) (331) (222)
Clarithromycin	- 12.52 w - - 20.87 vw 21.73 vw	**-** **-** **-** **-** **-** **-**	- - - - - -	- - - - - -	- - - - - -	- - - - - -	- - - - - -	- - - - - -	11.41 13.69 15.11 17.23 20.38 23.08	

w, weak; v, very; s, strong; m, intermediate. 1, El-Kahky et al. (2021); 2, Singh et al. (2024); 3, Ankegowda et al. (2020); 4, Samuggam et al. (2021); 5, Murtaza et al. (2024); 6, Lakhan et al. (2020); 7, Khan et al. (2022).

A detailed analysis of AgNPCA reveals an AgNP-XRD pattern with diffraction peaks at 2Theta values of 38.28°, 46.24°, 67.44°, and 77.0° corresponding to (111), (200), (220), and (311). These Bragg reflections belong to the lattice planes of face-centered cubic (fcc) Ag crystals according to JCPDS 04–0783 (Lakhan et al., 2020; Murtaza et al., 2024; Singh et al., 2023; Suvandee et al., 2022). Additionally, crystallographic planes of Ag2O related to fcc (JCPDS 041–1,104) are available at 27.8° (110), 32.24° (111), 37.9° (200), 54.86° (220), 61.72° (331), and 72.9° (222) (Figure 2; Table 2). The weak peaks in AgNPCA at 30.40°, and 29.04° are due to AgCl in agreement with previous reports (Figure 2; Table 2) (Al Aboody, 2019; Haj Bloukh et al., 2021). These compounds form after the addition of the antibiotic clarithromycin as by-products as a result to changes on the AgNP surface. After its addition, clarithromycin starts to compete with the clove-based compounds acting as stabilizing and capping agents on the AgNP surface. The antibiotic settles partly on the AgNP surface by dipol-dipol bonds between silver and its abundant oxygen atoms and compromises the monolayer on the AgNP surface. Furthermore, silver ions are released through oxidation and exchange processes during the equilibrium (Al Aboody, 2019; Reda et al., 2019; Singh et al., 2023). As a result, AgCl forms due to the availability of chloride ions in the clove bud extract and as inactive ingredient in clarithromycin itself, while Ag_2_O is formed increasingly through oxidation (Al Aboody, 2019; Reda et al., 2019; Singh et al., 2023).

The very weak bands around 5°–23.08° are due to semicrystalline, amorphous phases originating from clarithromycin and clove-based compounds within the sample (Figure 2; Table 2). Clarithromycin related weak to very weak peaks are seen in the red graph at 2Theta values of 12.52°, 20.87° and 21.73° in agreement with previous investigations (Figure 2; Table 2) (Khan et al., 2022).

As a conclusion, the sharp peaks in the XRD study of AgNPCA and AgNPC point to mainly crystalline AgNP with very limited semicrystalline, amorphous phases due to clove extract biomolecules and clarithromycin (Figure 2; Table 2). The overall XRD investigation confirms the purity of the nano-biohybrid AgNPC. Meanwhile in AgNPCA, AgCl and Ag_2_O emerge due to the addition of clarithromycin (Figure 2; Table 2).

### 3.4 UV-vis spectroscopy

**FIGURE 3:**
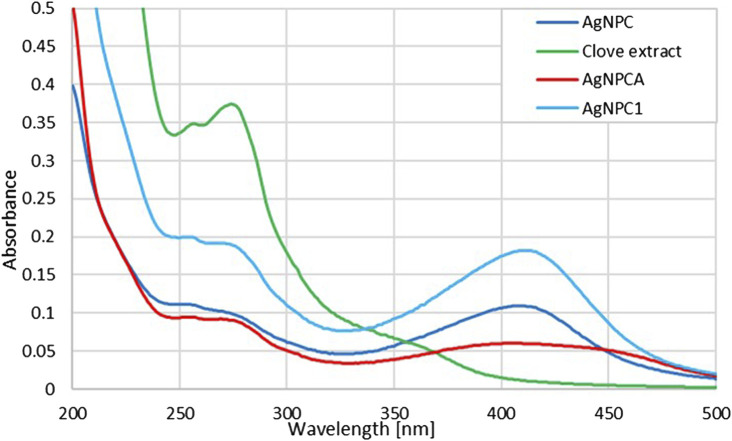
UV-vis analysis of AgNPCA, AgNPC, AgNPC1 and clove extract (200–500 nm). (AgNPCA: red; AgNPC: blue; AgNPC1: light blue; clove extract: green).

The UV-vis spectral analysis of clove extract, AgNPC, AgNPC1 and AgNPCA are presented in Figure 3.

Figure 3 provides insight into the changes of the clove extract during the AgNP formation (AgNPC). Furthermore, it presents the developments after introducing the antibiotic clarithromycin (AgNPCA). The phenolic compounds in the clove extract reduce the silver ions to metallic silver. The UV-vis spectrum can be used to verify these biocompounds in all the four samples (Figure 3; Table 3).

**TABLE 3:** UV-vis absorption signals in the samples clove extract, AgNPCA, AgNPC, AgNPC1, and further investigations [nm].

	Clove extract	AgNPCA	AgNPC	AgNPC1	1	2	3	4	5	6
	257 s 274 s 305 w, sh 364 w, sh	254 w, br 269 w, br 305 vw, sh 364^a^	251 w, br 269 w 305 vw, sh 364^a^	253 m, br 267 m, br 307 w, sh 364^a^	200 - 300 350	- - - -	206 - - -	- - 289 -	- 275 310 360	- - - -
AgNP	-	408 w, br	411 w, br	412 m, br	470–480	411	400–411	-	351	376

vw, very weak; br, broad; s, strong; m, intermediate; sh, shoulder; 1, Lakhan et al. (2020); 2, Mussin and Giusiano (2024); 3, Suvandee et al. (2022); 4, Murtaza et al. (2024); 5, Singh et al. (2024); 6, Ruban et al. (2023).

^a^ Broad bands cause overlapping, therefore peak cannot be located precisely.

The clove-based phenolic compounds appear in the region around 240–370 nm (Table 3). Therefore confirming availability of flavonoids, phenolic acids as hydroxybenzoic acids and hydroxycinnamic acids, as well as hydroxyphenyl propenes (Singh et al., 2023). The phenolic compounds are comprised of mainly eugenol, quercetin and kaempferol, ellagic acid, caffeic acid, as well as ferulic acid (Bloukh et al., 2020; Haj Bloukh et al., 2021; Singh et al., 2023). These compounds are verified in the green curve of clove extract by two main, strong absorption peaks at 257 and 274 nm, followed by a broad band at 364 nm and a weak shoulder at 305 nm (Figure 3; Table 3) (Bloukh et al., 2020; Haj Bloukh et al., 2021; Lakhan et al., 2020; Murtaza et al., 2024; Singh et al., 2023). The first three peaks at 257, 274 and possibly 305 nm can be attributed to flavonoids quercetin and kaempferol (flavonols), while the broad band at 364 nm indicates presence of phenolic acids (Bloukh et al., 2020; Haj Bloukh et al., 2021; Singh et al., 2023). The concerned phenolic compounds around 364 nm are related to eugenol, caffeic acid, ferulic acid and ellagic acid (Bloukh et al., 2020; Haj Bloukh et al., 2021; Lakhan et al., 2020; Murtaza et al., 2024; Singh et al., 2023) (Figure 3; Table 3).

The clove extract biocompounds absorption peaks at 257 and 274 nm are blue shifted towards shorter wavelengths in AgNPC (251 and 269 nm), AgNPC1 (253 and 267 nm), as well as in AgNPCA (254 and 269 nm) (Figure 3; Table 3). This hypsochromic effect underlines removal of conjugation and chromophores, solvent effect, saturation of -C=O to -C-O and an overall decreased size of the newly formed compounds compared to the clove extract components. AgNPC1 and AgNPCA were prepared by adding 10 mL of 10% AgNO_3_ into the clove extract, while AgNPC was based on 7.5 mL of a 10% AgNO_3_ solution into the clove extract. However, the best inhibitory results were achieved by AgNPC and AgNPCA as explained in the upcoming section below. Therefore, a smaller AgNP concentration in AgNPC leads to smaller NP size and better antimicrobial properties (Table 1). In comparison, higher Ag concentration in AgNPCA reveals better antimicrobial properties at larger NP size (Table 1). A second look at the undergoing change after adding clarithromycin reveals a red shift from 251 nm (AgNPC) to 254 nm (AgNPCA). The red curve of AgNPCA reveals also a shoulder at around 281 nm corresponding to clarithromycin itself in accordance to Pani and Chandrasekaran (2024)).

The UV-vis study shows the AgNP plasmonic peak at λ-max at 408, 411 and 412 nm for AgNPCA (red), AgNPC (blue) and AgNPC1 (light blue), respectively (Figure 3; Table 3). The surface plasmon resonance (SPR) absorbance band of AgNP undergoes two different shifts in this scenario. Adding clarithromycin into the sample AgNPC blue shifts the SPR band from 411 nm towards a broad band with a maximum at 408 nm in AgNPCA with a small shoulder at 450 nm. The blue shift confirms the capping of AgNPC by clarithromycin with its functional groups leading to aggregation (Adil et al., 2023). The same was reported by Adil et al. in their investigation of cephalosporins capped plant-based AgNP describing the phenomenon as accumulation of chemical groups around AgNP (Adil et al., 2023). The weak shoulder at 450 nm could be due to the aggregation and coating of AgNPC by organic molecules (Adil et al., 2023).

Additionally, the UV-vis spectrum shows a reduction of the AgNP peak starting from AgNPC1 to AgNPC and finally AgNPCA. The reduction of the AgNP peak indicates an increased coating of the AgNP surface by the available organic compounds in the solution, while resulting in a reduction of free AgNP (Pereira et al., 2024; Zúñiga-Miranda et al., 2023). However, once AgNPCA is formed by the addition of clarithromycin, the antibiotic induces the removal of the monolayer on the AgNPC and allows secondary nucleation. Organic molecules within the solution start coating the AgNP surface and lead to bigger sized aggregates coupled with a red shift (Table 1). Clarithromycin induces the removal of the monolayer on the AgNPC and allows secondary nucleation. Organic molecules within the solution start coating the AgNP surface and lead to bigger size coupled with a red shift (Table 1) (Ormeño-Martínez et al., 2024; Pereira et al., 2024; Samari-Kermani et al., 2021; Ullah et al., 2023; Yang et al., 2017; Zúñiga-Miranda et al., 2023). This red shift verifies the DLS measurements and the XRD results by underscoring the increase in AgNP size (Tables 1, 2).

The SPR band is in accordance with previous investigations. Mussini et al. and Suvandee et al. had an AgNP SPR band around 411 nm, while other studies reported lower wavelengths between 350 and 400 nm (Lakhan et al., 2020; Mussin and Giusiano, 2024; Samuggam et al., 2021; Suvandee et al., 2022). Our previous investigations of plant-based synthesis of AgNP with *Cinnamomum zeylanicum* and *Lepidium sativum* resulted in SPR bands around 390-415 and 400 nm, respectively (Bloukh et al., 2020; Haj Bloukh et al., 2021). The pH levels in both studies were around 8.5, with a silver ion concentration of 10%, while in the synthesis of AgNPC, the best results were achieved with 30% silver ion concentration and a pH of 8.5 (Bloukh et al., 2020; Haj Bloukh et al., 2021). In general, the SPR band shape and values depend on the surrounding environment, stabilizing agents, method of synthesis, size morphology and further factors (Desai et al., 2023).

### 3.5 Fourier-transform infrared (FTIR) spectroscopy

**FIGURE 4:**
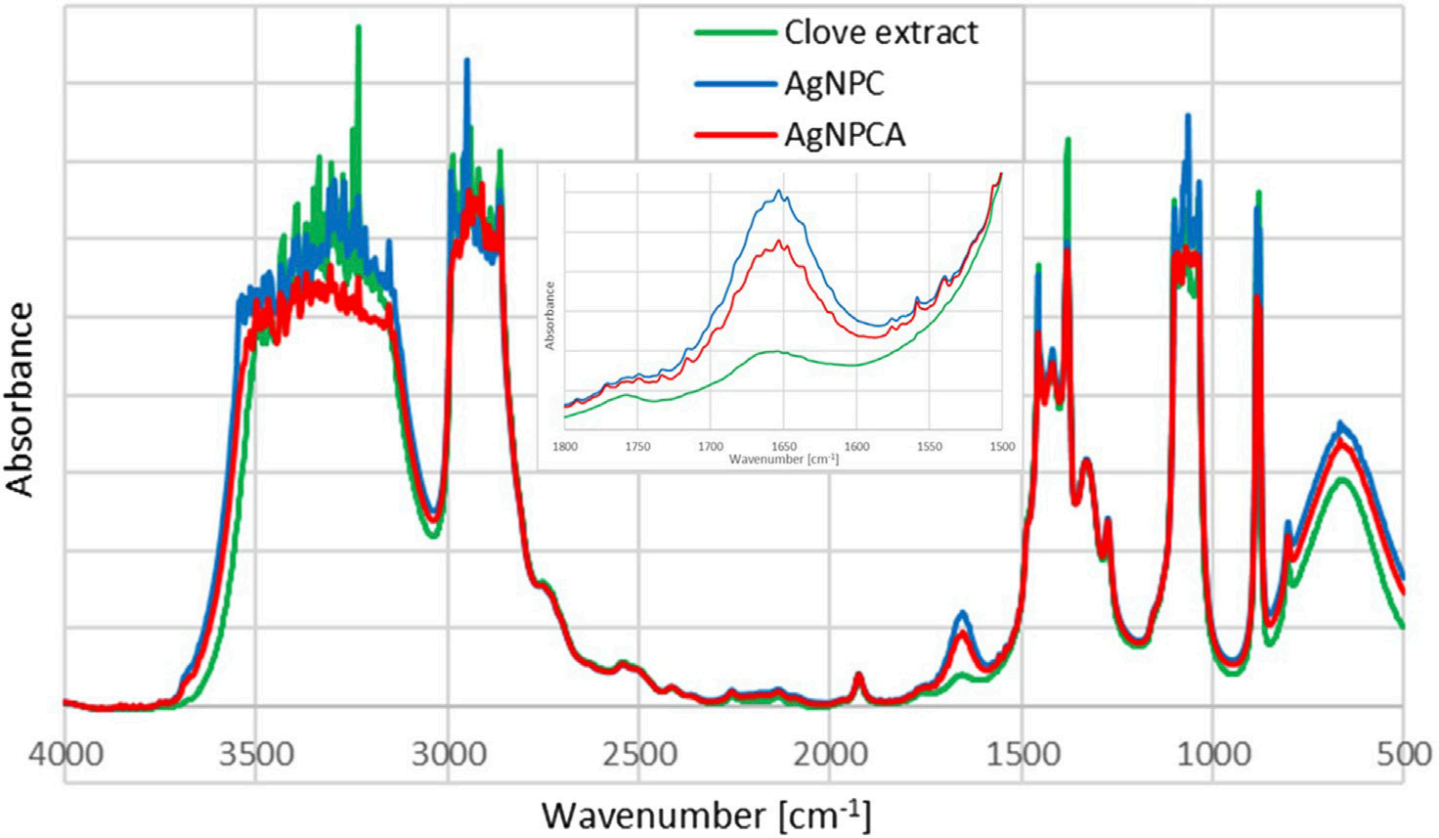
Fourier-Transform Infrared (FTIR) spectroscopic analysis of AgNPCA (red), AgNPC (blue) and pure clove extract (green).

The FTIR analysis of AgNPCA, AgNPC and pure clove extract reveals purity and similar structural features within the samples (Figure 4).

The FTIR analysis of the title compounds AgNPC (blue) and AgNPCA (red), together with the clove extract (green) in Figure 4 allow insight into the changes during nanoparticle formation and antibiotic addition. Both nanoparticle formulations have similar pattern in the FTIR spectrum and are in accordance with previous reports (Figure 4) (Bloukh et al., 2020; Haj Bloukh et al., 2021). In general, the FTIR study reveals highest absorption intensities for the clove extract (green), followed by AgNPC (blue) and lastly AgNPCA (red) (Figure 4). This pattern is exempted in the regions around 1,647 cm^−1^, 1,100–1,000 cm^−1^ and 800–600 cm^−1^, which belong to vibrational stretching bands of -C=O, -C-O, as well as twisting- and bending vibrations of -CH_2_, -C-H and O-H groups (Figure 4; Table 4).

**TABLE 4:** FTIR analysis of AgNPCA, AgNPC, and clove extract in solvent ethanol [cm^–1^].

	ν_1,2_ (O–H)_s,a_ ν (COOH)_a_	ν (C–H)_a_	ν (C-H)_s_	ν (C=O)_a_	δ (C-H)_a_ δ (CH_2_) δ (O-H)	ν (C-C)	ν (C-O)	ν (C-O) ν (C-N)
AgNPCA	3497 s 3466 s 3369 s 3305 vs 3232 s 3152 s	2988 sh 2982 s 2941 s	2909 s 2889 s 2862 s	1749 vw 1730 vw 1717 vw 1647 w,br 1570 w 1574 vw 1531 w 1506 m	1456 m δ(CH_3_)_s, in-plane_ 1417 m δ(CH_3_)_a, in-plane_ 883 s δ(CH_2_)_twisting_ 878 s δ(CH_2_)_twisting_ 802 m δ(C-H)_out-of-plane_ 667 s δ(O-H)	1379 s 1321 m	1271 m	1154 w,sh ν (C-N) 1101 s ν (C-O) 1092 s ν (C-O) 1070 vs ν (C-O) 1065 s ν (C-O) 1055 s ν (C-O) 1051 s ν (C-O) 1047 s ν (C-O) 1042 s ν (C-O) 1038 s ν (C-O)
AgNPC	3497 s 3466 s 3368 s 3305 vs 3232 s 3152 s	2989 s 2982 s 2947 vs	2909 s 2889 s 2862 s	1749 vw 1730 vw 1717 vw 1647 w,br 1570 w 1574 vw 1531 w 1506 m	1456 s δ(CH_3_)_s, in-plane_ 1417 m δ(CH_3_)_a, in-plane_ 885 vs δ(CH_2_)_twisting_ 878 s δ(CH_2_)_twisting_ 802 m δ(C-H)_out-of-plane_ 667 s δ(O-H)	1379 s 1321 m	1271 m	1154 w,sh ν (C-N) 1099 vs ν (C-O) 1092 s ν (C-O) 1072 vs ν (C-O) 1065 vs ν (C-O) 1055 s ν (C-O) 1051 s ν (C-O) 1043 s ν (C-O) 1042 s ν (C-O) 1036 vs ν (C-O)
Clove extract	3478 s 3399 s 3334 vs 3232 s	2986 vs 2978 s 2945 s	2909 s 2889 s 2862 vs	1647 vw,br	1456 s δ(CH_3_)_s, in-plane_ 1417 m δ(CH_3_)_a, in-plane_ 883 s δ(CH_2_ **)**_twisting_ 878 s δ(CH_2_)_twisting_ 802 m δ(C-H)_out-of-plane_ 667 s δ(O-H)	1379 vs 1321 m	1271 m	1154 w,sh ν (C-N) 1101 vs ν (C-O) 1070 vs ν (C-O) 1051 s ν (C-O) 1036 s ν (C-O)

ν, vibrational stretching; δ, deformation; s, symmetric; a, asymmetric; absorption intensity: vs, very strong; s, strong; m, medium, vw, very weak; sh, shoulder; red color, red shift from AgNPC to AgNPCA; blue color, blue shift from AgNPC to AgNPCA and yellow highlighting, not available in clove extract (C).

The mentioned exceptions in absorption intensity point towards developments triggered by AgNP synthesis and addition of clarithromycin (Figure 4). The concerned functional groups in AgNPC absorb more with high intensity, because they are less encapsulated/complexed by hydrogen bonding. Once clarithromycin is added, hydrogen bonding is enabled between the many functional groups of the antibiotic with the already existing clove extract phenolic compounds, flavonoids, the solvent and the AgNP surface. All together reduce the absorption intensity of AgNPCA. These affected structural parts of the AgNPCA molecules are vibrational stretching bands of -C=O at 1,647 cm^−1^, as well as -C-O at 1,101, 1,070, 1,065, 1,055, 1,047 and 1,038 (Figure 4; Table 4). Additionally, vibrational twisting bands of -CH_2_ at 883 and 878 cm^−1^, as well as out-of-plane bending vibrations of -CH at 802 cm^−1^ and bending vibrations of -OH at 667 cm^−1^. The three latter seem to undergo complexation or encapsulation processes throughout the molecules, seemingly decreasing their interatomic distances. Therefore, the twisting/out-of-plan/bending motions of the methylene-, -C-H, as well as the hydroxyl-groups are characterized by reduced flexibility and are not free enough to interact with the IR light.

The vibrational stretching band at 1,647 cm^−1^ related to -C=O bonds has the highest intensity for AgNPC, followed by AgNPCA and the clove extract. The increased absorbance of carbonyl stretching vibrations in AgNPC is the result of an increase in conjugation systems and chromophores after adding silver ions into the clove extract. The increase in intensity verifies the formation of Ag-O interactions between the metallic silver in AgNP and the surrounding biocomponents. The biocompounds with -C-OH groups in the clove extract, including eugenol are oxidized to -C=O and reduce the silver ions to metallic silver nanoparticles. During this process, the phenolic compounds and flavonoids act as capping and stabilizing agent on the silver surface, forming a monolayer, preventing secondary nucleation. Furthermore, this action prevents agglomeration and keeps the size of AgNP in AgNPC small verifying the results in the DLS analysis. Once clarithromycin is added into the compound, AgNPCA is formed. AgNPCA has lower absorption intensities for carbonyl- (1,647 cm^−1^) and -C-O (1,099–1,036 cm^−1^) stretching vibrations in comparison to AgNPC. Here, the monolayer is compromised by clarithromycin, which competes and interacts with the other biocompounds on the Ag surface. Accordingly, there is a breach in the monolayer, which leads to release of silver-ions, formation of AgCl and Ag_2_O (Al Aboody, 2019; Haj Bloukh et al., 2021; Singh et al., 2024). Additionally, secondary nucleation steps in leading to increase of AgNP size through agglomeration, which is also verified by DLS studies and UV-vis analysis. Similar processes influence the twisting- and bending vibrations of methylene, -CH and hydroxyl groups around 800–600 cm^−1^ (Figure 4; Table 4). The absorption bands for these groups also show lower intensities in AgNPCA due to the increased hydrogen bonding between clarithromycin and AgNPC.

AgNPC and AgNPCA absorption bands between 3,500 and 3,100 cm^−1^ are related to -COOH and -OH groups. Both spectra show a slight broadening compared to the clove extract indicating more hydrogen bonding for these groups (Figure 4; Table 4). Both AgNP samples contain new, very strong vibrational bands at 3,305 and 3,125 cm^−1^, which are lacking in the clove extract (Table 4, yellow highlighted). The region for asymmetric and symmetric vibrational -C-H stretching bands between 3,000 and 2,860 cm^−1^ contains interesting details. The FTIR spectra of AgNPC displays two very strong vibrational asymmetric stretching bands for -C-H bonds at 2,989 and 2,947 cm^−1^ (Figure 4; Table 4). These two are red shifted towards 2,988 and 2,941 cm^−1^ with lower absorption intensity in AgNPCA. Therefore, a weakening of asymmetric -C-H bonds due to the addition of clarithromycin is expected. The same happens to the very strong vibrational stretching bands of -C-O at 1,072 cm^−1^ in AgNPC, which are red shifted in AgNPCA to 1,070 cm^−1^ (Figure 4; Table 4). After adding clarithromycin, blue shifts as a marker for stronger bonds and encapsulation for the concerned -C-O stretching bands are present in AgNPC at 1,099, 1,043 and 1,036 cm^−1^ towards AgNPCA at 1,101, 1,047 and 1,038 cm^−1^, respectively (Figure 4; Table 4).

Ullah et al. detected in the FTIR analysis alcohol O-H stretching bands at 3,744.1 cm^−1^, carboxylic acid O-H stretching bands at 2,961.4 cm^−1^, alkane C-H bending at 1,462.2, and bands related to alcohol C-O stretching at 1,012.1 cm^−1^ (Ullah et al., 2023). AgNPCA reveals in the FTIR spectrum similar related bands at 3,694.9, 2,943.4, 1,456.3 and 1,039.6 cm^−1^, respectively (Figure 4). All these bands are in comparison to pure clarithromycin red shifted, except for the alcohol C-O stretching (Ullah et al., 2023). The red shifts indicate an increase in conjugation systems for the related groups, while the only small blue shift of the alcohol C-O stretching maybe due to solvent effect. As a result, the addition of clarithromycin leads to an increase in conjugation systems by oxidation of alcoholic groups on clarithromycin (Ullah et al., 2023).

The FTIR spectrum of pure clove extract contains eugenol as one of its main ingredients (Hameed et al., 2021). The bands at 3,369 cm^−1^ (-OH), 2,982 cm^−1^ (-C-H), 1,647 cm^−1^ (-C=O), 1,456 cm^−1^ (-CH_3_), 1,154 cm^−1^ (-C-N), 1,051 cm^−1^ (-C-O) can be assigned to eugenol according to previous studies (Hameed et al., 2021; Mohammed at el., 2021; Murtaza et al., 2024).

**FIGURE 5:**
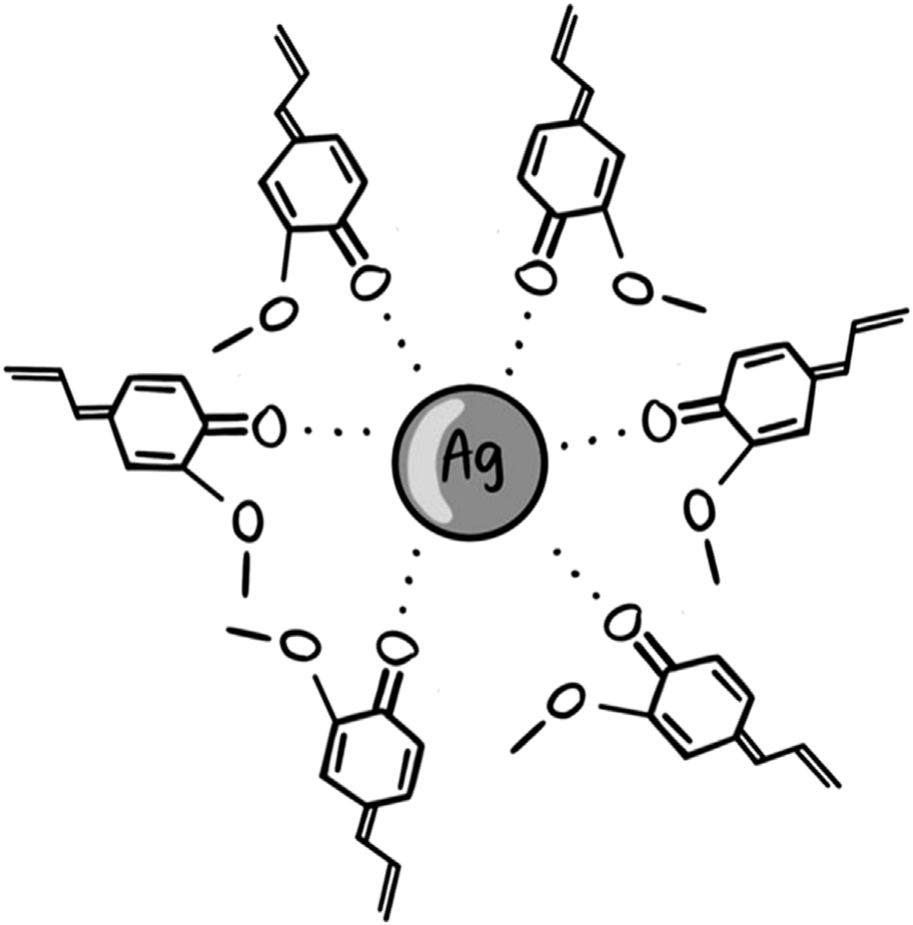
Proposed illustration for AgNPC stabilized by oxidized eugenol molecules.

In conclusion, the increase in absorption intensity of C=O groups at 1,647 cm^−1^ from clove extract to AgNPC verifies the synthesis of AgNP through oxidation of hydroxyl groups in the phenolic compounds and flavonoids. These biocompounds form Ag---O=C interactions and stabilize or cap the metallic Ag surface, establishing a monolayer and preventing agglomeration (Figure 5).

Once clarithromycin is introduced into the system, the capping agents are degraded, destabilized, leading to a lower acetylation degree (Haj Bloukh et al., 2021).

### 3.6 Antimicrobial activities of AgNPC and AgNPCA

Disc diffusion assay (DD) was utilized in order to check the title compounds AgNPC and AgNPCA effect on ten reference strains. The selected panel consisted of Gram-positive bacteria *S. pneumoniae* ATCC 49619*, S. aureus* ATCC 25923*, S. pyogenes* ATCC 19615, *E. faecalis* ATCC 29212 and *B. subtilis* WDCM0003, as well as the Gram-negative *E. coli* WDCM 00013 Vitroids*, P. mirabilis ATCC 29906*, *P. aeruginosa* WDCM 00026 Vitroids and *K. pneumoniae* WDCM00097 Vitroids) and one fungus type (*C. albicans* WDCM 00054 Vitroids).

The antimicrobial testing results on positive control antibiotics (A), clove extract (1), AgNPC (0.81 μg/mL), AgNPC1 (1.08 μg/mL) (2), AgNPCA (1.08 μg/mL), AgNPCA1 (0.81 μg/mL) (4) and clarithromycin solution (1.34 μg/mL) (5) are presented in Table 5.

**TABLE 5:** Antimicrobial testing of antibiotics (A), clove extract (1), AgNPC, AgNPC1 (2) AgNPCA (3), AgNPCA1 (4) and clarithromycin solution (5). ZOI (mm) against microbial strains by disk diffusion (DD) assay.

Strain	Antibiotic	A	1	AgNPC	2	AgNPCA	4	5
*S. aureus* ATCC 25923	G	28	7	25	12	25	25	25
*E. faecalis* ATCC 29212	G	25	R	20	17	15	R	16
*S. pyogenes* ATCC 19615	G	25	R	17	18	16	R	17
*S. pneumoniae* ATCC 49619	G	18	R	18	18	15	R	17
*B. subtilis* WDCM 00003	G	21	R	10	R	30	30	25
*P. mirabilis* ATCC 29906	G	30	R	20	R	R	R	R
*E. coli* WDCM 00013	G	30	R	15	7	11	12	R
*P. aeruginosa* WDCM 00026	G	23	R	8	10	7	11	R
*K. pneumoniae* WDCM 000097	G	30	R	R	12	12	13	R
*C. albicans* WDCM 00054	NY	16	R	R	R	R	R	R

Disc diffusion studies (6 mm disc impregnated with 2 mL of clove extract (5 μg/mL) (1), AgNPC (0.81 μg/mL), AgNPC1 (1.08 μg/mL) (2), AgNPCA (1.08 μg/mL), AgNPCA1 (0.81 μg/mL) (4) and clarithromycin (1.34 μg/mL) (5). A = Gentamicin (G, 30 µg/disc). Nystatin (NY, 100 IU). The grey shaded area represents Gram-negative bacteria. 0 = Resistant. No statistically significant differences (*p* > 0.05) between row-based values through Pearson correlation.

**FIGURE 6:**
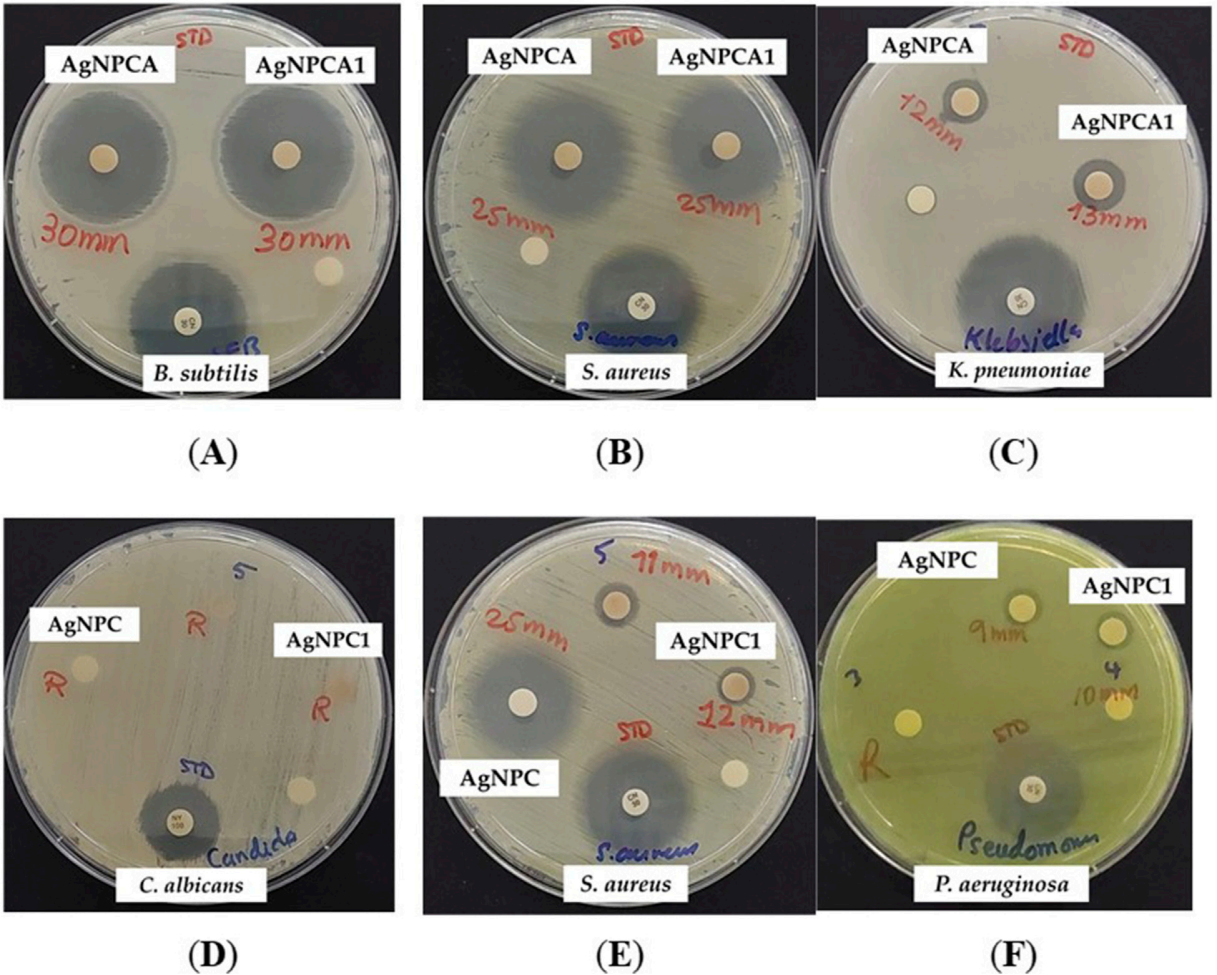
AgNPCA and AgNPC coated sterile discs (disc diffusion assay) with positive control antibiotic nystatin (100 IU) and gentamicin (30 µg/disc). From left to right: AgNPCA (1.08 μg/mL) against **(A)** *B. subtilis* WDCM 00003; **(B)** *S. aureus* ATCC 25932; **(C)** *K. pneumoniae* WDCM 00097; AgNPC (0.81 μg/mL) against **(D)** *C. albicans* WDCM 00054; **(E)** *S. aureus* ATCC 25932; **(F)** *P. aeruginosa* WDCM 00026.

Table 5 signifies the vulnerability of 9 microorganism strains towards the two formulations AgNPC and AgNPCA. When the results of both formulations are compared, it is noted that AgNPC generally has better results, except in two strains (Gram-positive *B. subtilis* WDCM 00003 and Gram-negative *K. pneumoniae* WDCM 000097) (Table 5). The results show a lower reaction to *B. subtilis* WDCM 00003 (10 mm) and a complete resistance against *K. pneumoniae* WDCM 000097, which indicates that the formulation with the antibiotic clarithromycin worked better against those 2 strains (Table 5). The results were almost similar in both formulations against *S. aureus* ATCC 25923, *S. pyogenes* ATCC 19615, and *P. aeruginosa* WDCM 00026. *C. albicans* WDCM 00054 shows resistance to all tests done except to the positive control antibiotic nystatin with a result of 16 mm (Table 5). Interestingly, when the antibiotic clarithromycin is present (AgNPCA) resistance is observed in *P. mirabilis* ATCC 29906, while in AgNPC the resistance is resolved and an inhibition zone of 20 mm is seen instead (Table 5). The opposite is detected for *K. pneumoniae* WDCM 000097, where the resistance was in AgNPC, and an inhibitory zone of 12 mm in AgNPCA (Table 5). Clove extract showed resistance to all strains except *S. aureus* ATCC 25923, where it had a result of 7 mm (Table 5). Similarly, clarithromycin diluted in ultradistilled water was resistant to all negative bacterial strains, but when added into the formulation showed good results. This implies that the addition of clove extract and silver nanoparticles helped overcome the resistance by working synergistically (Table 5; Figure 6).

AgNPC inhibits all gram-positive and Gram-negative strains except *K. pneumoniae* WDCM 000097 and the fungus *C. albicans* WDCM 00054 (Table 5; Figure 6). Comparatively, AgNPCA inhibited all Gram-positive bacteria and some Gram-negative excluding *P. mirabilis* ATCC 29906 and *C. albicans* WDCM 00054 (Table 5; Figure 6). Furthermore, the highest inhibition of strains occurs in the AgNPCA formulation against *B. subtilis* WDCM 00003 (30 mm), followed by *S. aureus* ATCC 25923 (25 mm) seen in both AgNPC and AgNPCA (Table 5; Figure 6).

Previous studies with plant biosynthesized AgNP display mixed results (Bloukh et al., 2020; Haj Bloukh et al., 2021). In comparison, our studies of *Lepidium Sativum* L. based AgNP (LS-AgNP-1.08 μg/mL) with the same set of 10 reference strains achieved ZOI of 20 mm for Gram-negative *P. aeruginosa* WDCM 00026, 15 mm for *E. coli* WDCM 00013 and *K. pneumoniae* WDCM 000097 (Haj Bloukh et al., 2021). However, Gram-positive *S. pneumoniae* (15 mm), *S. aureus* ATCC 25923 (14 mm), *S. pyogenes* ATCC 19615 (13 mm), and *E. faecalis* ATCC 29212 (13 mm) were less susceptible towards LS-AgNP (Haj Bloukh et al., 2021). In another study, we investigated AgNP through trans-cinnamic acid (TCA) and *Cinnamomum Zeylanicum* (Cinn) (Bloukh et al., 2020). Under the set of 10 reference strains, TCA-AgNP performed even better than LS-AgNP and Cinn-AgNP (Bloukh et al., 2020). At a concentrations of 50 μg/mL, TCA-AgNP exerted antifungal properties against *C. albicans* WDCM 00054 (Bloukh et al., 2020). Therefore, we reported, that the antifungal properties do not originate from AgNP, nor Cinn extract (Bloukh et al., 2020). Singh et al. investigated their Ag-Fe bimetallic nanoparticles based on clove bud extract (Singh et al., 2024). Their Agar-Well (AW) diffusion tests revealed ZOI = 11, 9.3 and 10 mm against *S. aureus*, *E. coli* and *P. aeruginosa* (Singh et al., 2024). AW studies usually achieve higher ZOI, because the sample is directly poured in a well inside the petri-dish. In our studies, we used DD methods, which first require dip-coating and then drying the disks at ambient temperature. Accordingly, DD studies may record smaller inhibitory zones. Further recent investigations used clove buds and clove powder extracts for the biosynthesis of AgNP and/or Ag-FeNP and reported similar results (Lakhan et al., 2020; Murtaza et al., 2024).

As a result, discs impregnated with the two formulations exhibit somewhat similar promising antibacterial activities with a few variations such as the resistance mentioned with *P. mirabilis* ATCC 29906 against AgNPCA and the resistance observed in *K. pneumoniae* WDCM 000097 against AgNPC. *C. albicans* WDCM 00054 was not susceptible to AgNPC and AgNPCA (Table 5; Figure 6).

Further interesting results were demonstrated by AgNPCA against *B. subtilis* WDCM 00003 (30 mm), followed by Gram-positive *S. aureus* ATCC 25923 (25 mm), *S. pyogenes* ATCC 19615 (16 mm), *E. faecalis* ATCC 29212 (15 mm) and *S. pneumoniae* ATCC 49619 (15 mm), Gram-negative *K. pneumoniae* WDCM 000097 (12 mm), *E. coli* WDCM 00013 (11 mm), *P. aeruginosa* WDCM 00026 (7 mm) (Table 5; Figure 6).

AgNPC showed the best results in *S. aureus* ATCC 25923, *E. faecalis* ATCC 29212 and *P. mirabilis* ATCC 29906, *S. pneumoniae* ATCC 49619, *S. pyogenes* ATCC 19615, *E. coli* WDCM 00013, *B. subtilis* WDCM 00003, and lastly *P. aeruginosa* WDCM 00026 (Table 5; Figure 6).

Additional *in vivo*, as well as toxicity studies are needed to confirm the potential use of AgNPC and AgNPCA as antibacterial agents.

## 4 Conclusion

Antimicrobial resistance is a fatal threat to human health, causing drastic changes in the medical field. Plant based alternatives have been proven by countless studies to contain components that have antimicrobial, antiviral, antioxidant and antifungal properties. The combination of plant-based AgNP with antibiotics, here clarithromycin, offers possible solutions to existing problems. Synergistic effects between these compounds could increase antimicrobial properties, reduce the needed dosage of antibiotic, mitigate toxicity and AMR. This study shed light to the potential of the title compounds AgNPC and AgNPCA as antibacterial agents.

AgNPCA revealed synergistic action between the antibiotic clarithromycin, AgNP and clove extract. In this regard, *B. subtilis* WDCM 00003 and *S. aureus* ATCC25923 were higly susceptible with 30 and 25 mm ZOI towards AgNPCA, respectively. As a result, the studied Gram-negative pathogens were susceptible towards AgNPCA. Clarithromycin alone does not inhibit any Gram-negative pathogen. However, the presence of clarithromycin in AgNPCA removed partly the stabilizing capping agents consisting of phenolic compounds and flavonoids from the clove extract.

The stability of AgNPC is confirmed by DLS analysis through a suitable nanoparticle size with negative zeta potential. However, adding clarithromycin, a relatively big, macrocyclic compound in AgNPCA increases size and zeta potentials. The electrostatic interactions between clarithromycin functional groups with the capping agents and the Ag surface in AgNPCA resulted in secondary nucleation, partly release of capping agents and silver ions. The EDS of AgNPCA confirmed the availability of AgCl and Ag_2_O as a result of the release of silver ions. The DLS analysis reported increase of nanoparticle size and instability of the NP in the colloidal solution when clarithromycin was added resulting in AgNPCA. However, the impact of steric stabilization due to clarithromycin and clove biocompounds seem to counterbalance agglomeration. A comparison between SEM and DLS size measurements reveals the formation of a stabilizing organic layer around the nanoparticles.

AgNPC achieved the best disc diffusion results with ZOI = 20 mm against the Gram-positive strains *S. aureus* ATCC 25923 and *E. faecalis* ATCC 29212, as well as the Gram-negative, highly motile *P. mirabilis* ATCC 29906. *S. pneumoniae* ATCC 49619, a known resistant pathogen was susceptible towards the title compound AgNPC with 18 mm on the same level of gentamycin (positive control). AgNPC and AgNPCA have shown to have promising results at low concentrations but failed to overcome the resistance caused by *C. albicans* WDCM 00054. This pathogen is not susceptible to AgNP nor a low concentration of biocompounds from plant extracts.

As a conclusion, the clove extract-based biosynthesis of silver nanoparticles resulted in small sized, stable AgNPC with almost homogenous morphology and high purity. The increase in the nanoparticle size was not detrimental for inhibitory action of AgNPCA against Gram-negative pathogens in comparison to pure clarithromycin and AgNPC. Pathogens resistant against the heterocyclic antibiotic clarithromycin were inhibited by AgNPCA. Further *in vivo* and cytotoxicity studies are necessary to verify the use of AgNPC and AgNPCA as antibacterial agents.

## Data Availability

The original contributions presented in the study are included in the article/Supplementary Material, further inquiries can be directed to the corresponding author.

## Abbreviations

AMR, Antimicrobial Resistance; AgNP, Silver nanoparticle; C, Clove extract; A, Antibiotic Clarithromycin; EDS, Energy Dispersive Spectroscopy; FTIR, Fourier transmission infrared spectroscopy; Raman, Raman spectroscopy; SEM, Scanning electron microscopy; UV-vis, Ultraviolet-visible spectroscopy; WHO, World Health Organization; XRD, X-ray crystallography; ZOI, Zone of Inhibition.
