# Supplementary figures and images for: Syzygium aromaticum extract mediated, sustainable silver nanoparticle synergetic with heterocyclic antibiotic clarithromycin and their antimicrobial activities
Source: Front Chem. 2025 Jan 15;12:1513150. doi: 10.3389/fchem.2024.1513150 (PMC11775004; doi:10.3389/fchem.2024.1513150)

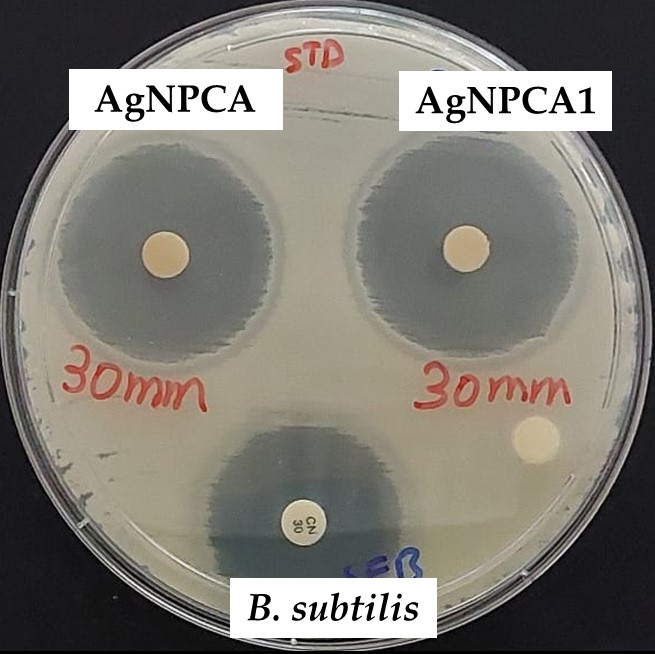

Supplement: Supplementary file 1 [file Image3.jpeg]

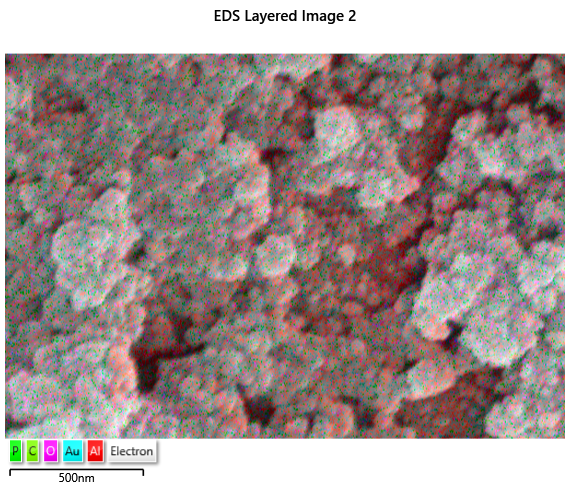


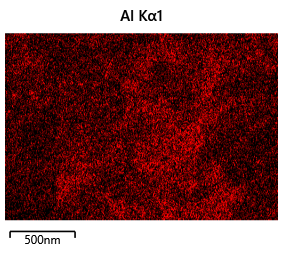

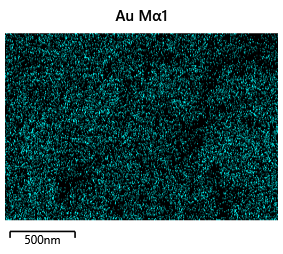

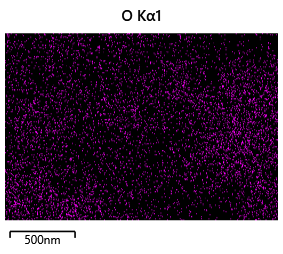

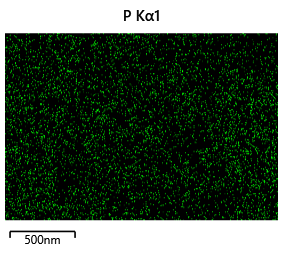

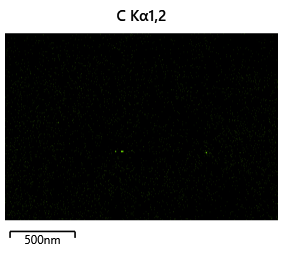

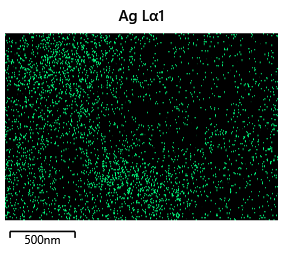

Supplement: Supplementary file 2 [file Supplementaryfile1.docx]

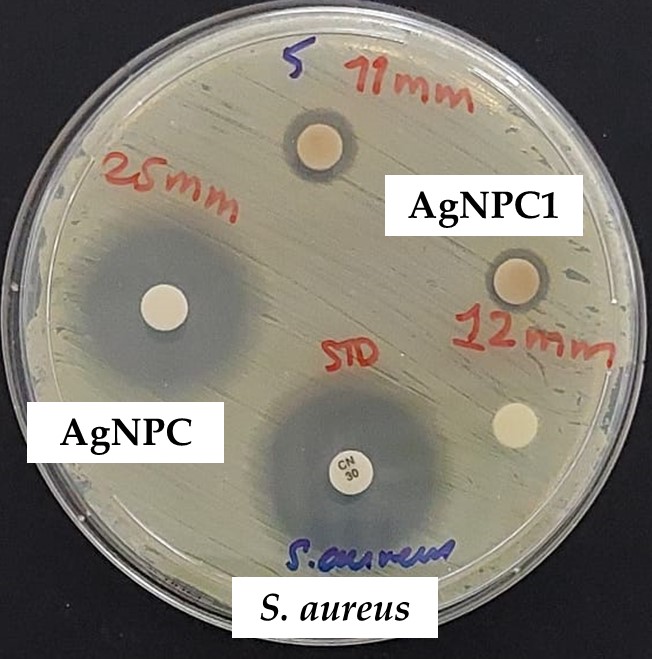

Supplement: Supplementary file 3 [file Image1.jpeg]

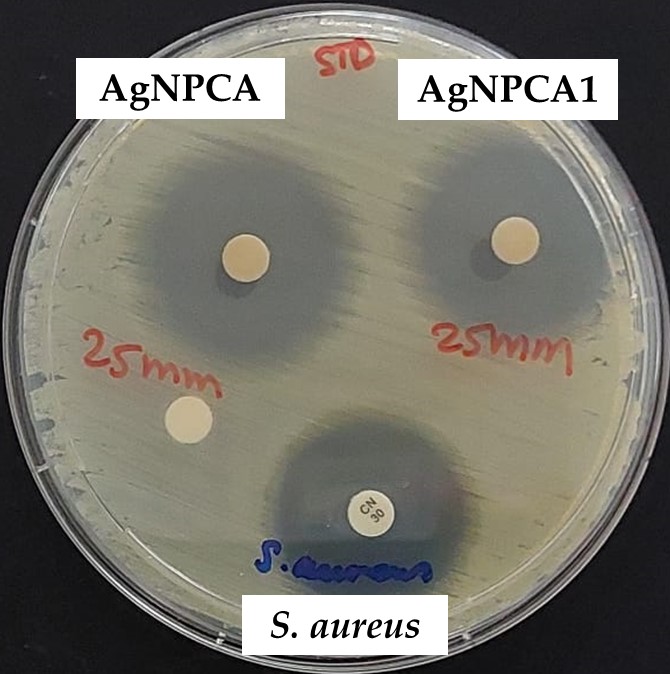

Supplement: Supplementary file 4 [file Image4.jpeg]

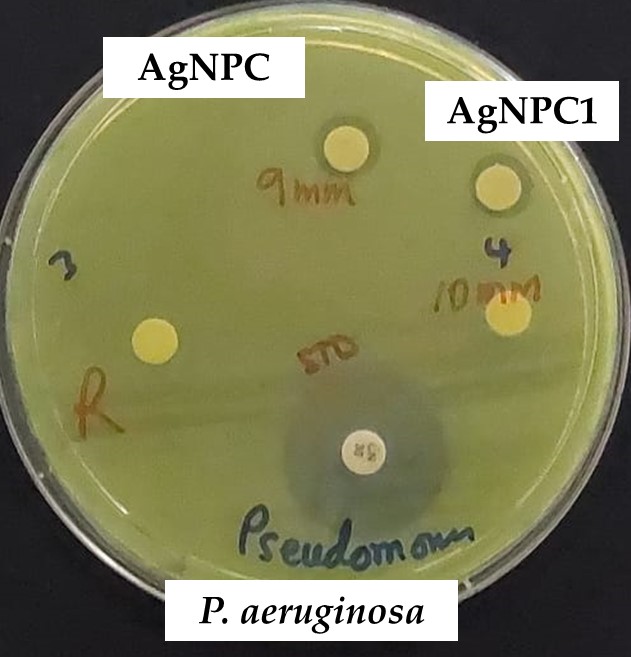

Supplement: Supplementary file 6 [file Image2.jpeg]

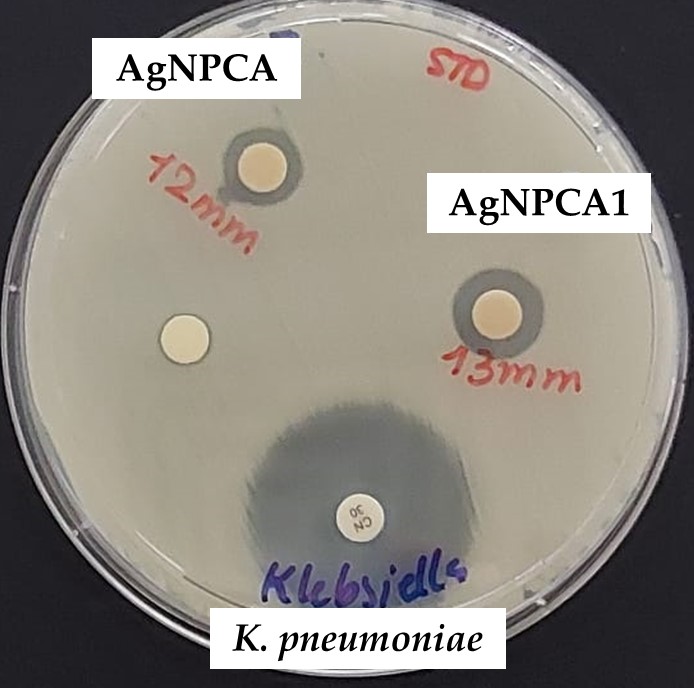

Supplement: Supplementary file 7 [file Image5.jpeg]

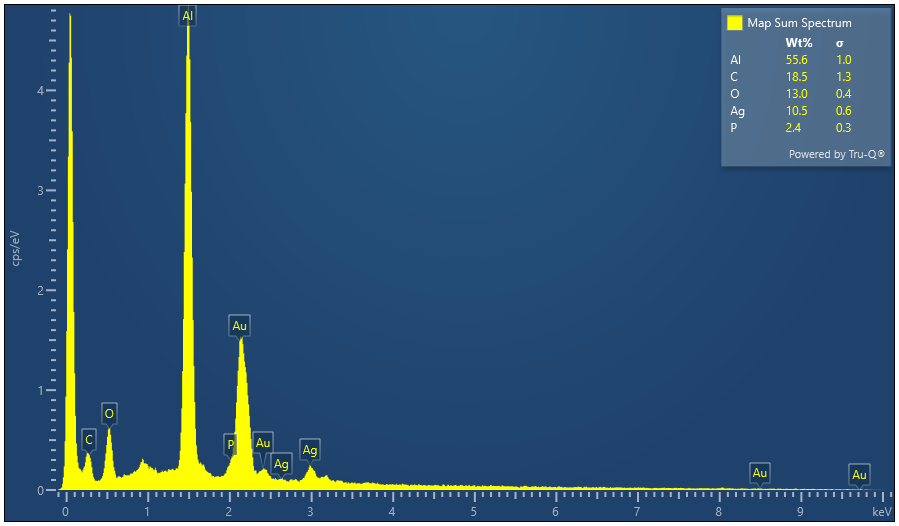

Supplement: Supplementary file 8 [file Supplementaryfile3.docx]

# SAMPLE 1

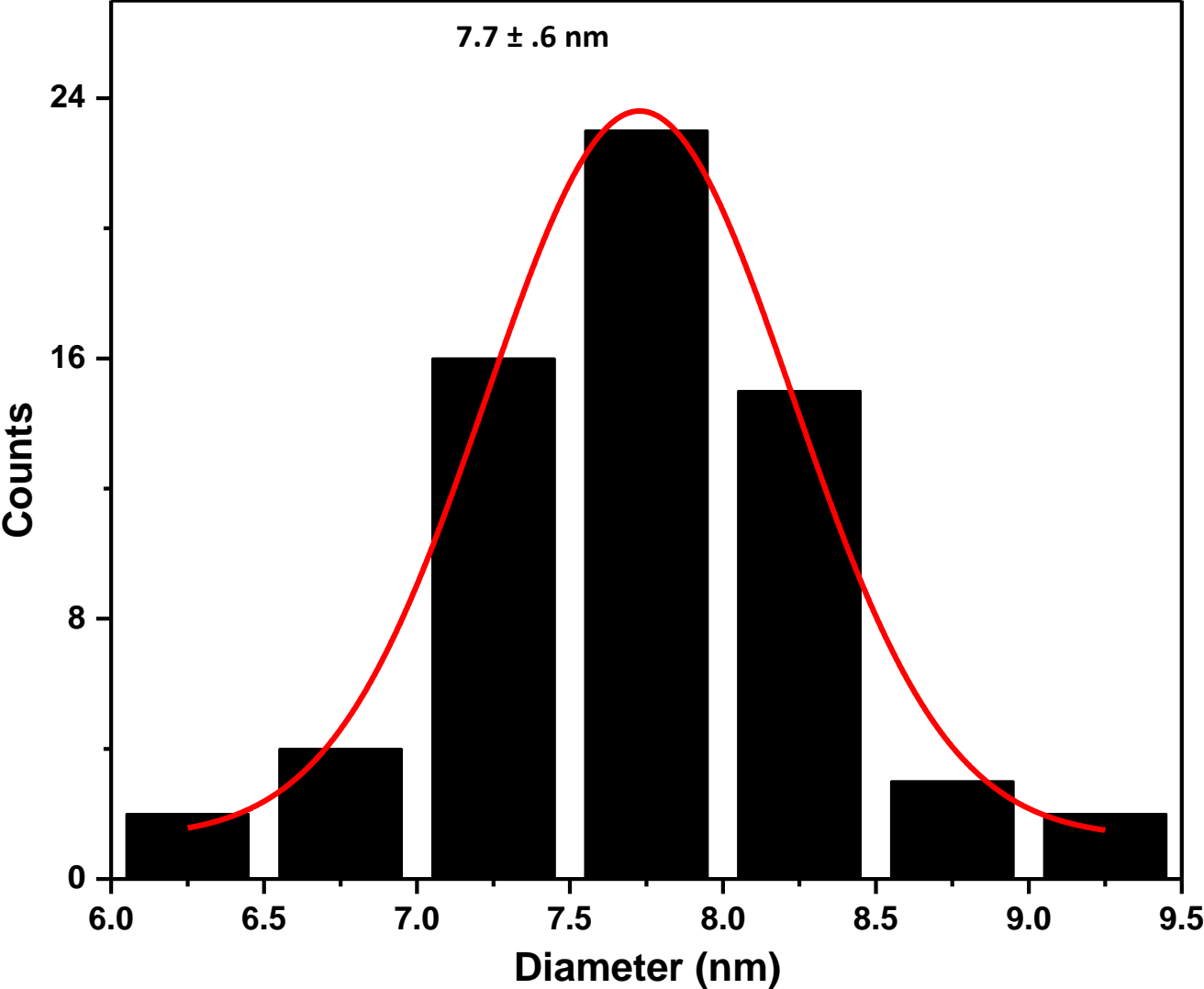

**SAMPLE 3**

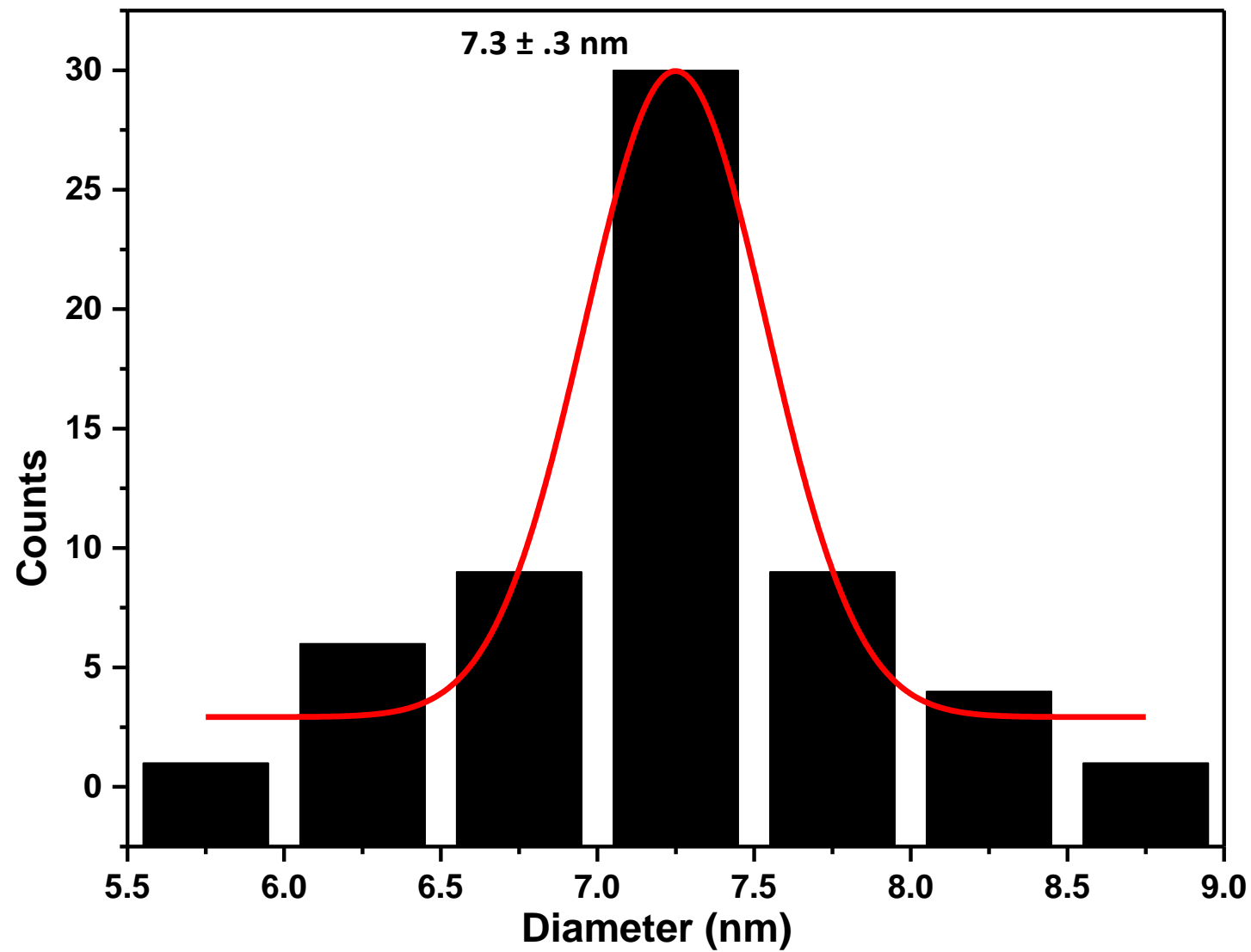

Supplement: Supplementary file 9 [file DataSheet1.pdf]

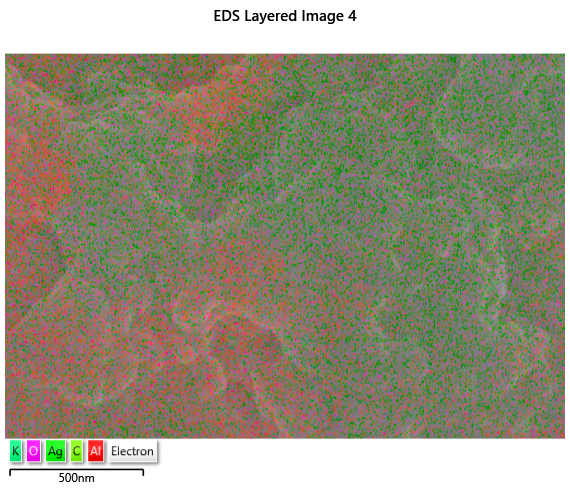


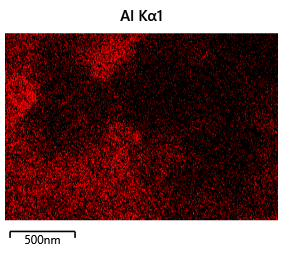

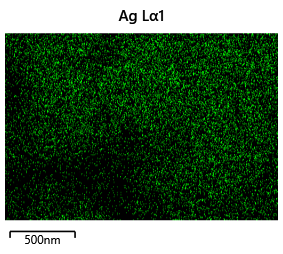

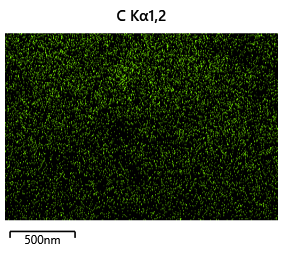

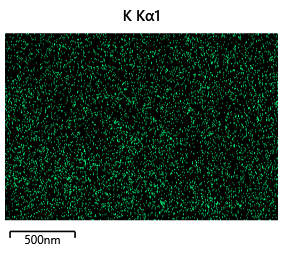

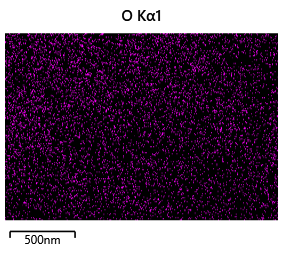

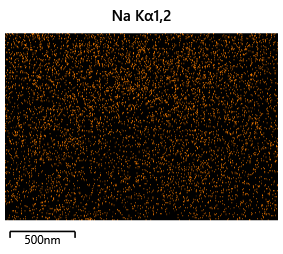

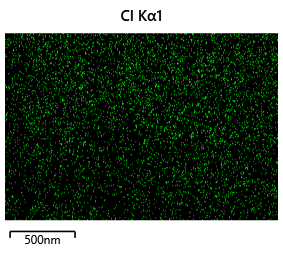

Supplement: Supplementary file 10 [file Supplementaryfile2.docx]

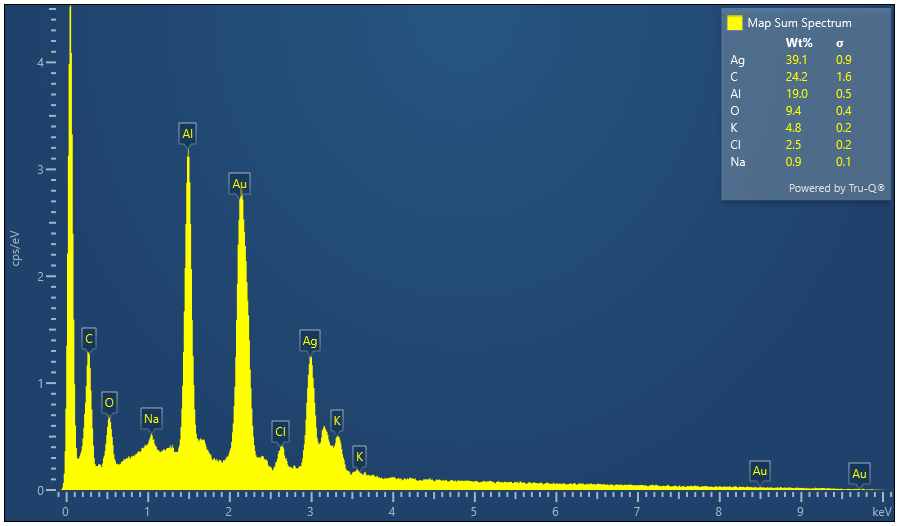

Supplement: Supplementary file 11 [file Supplementaryfile4.docx]

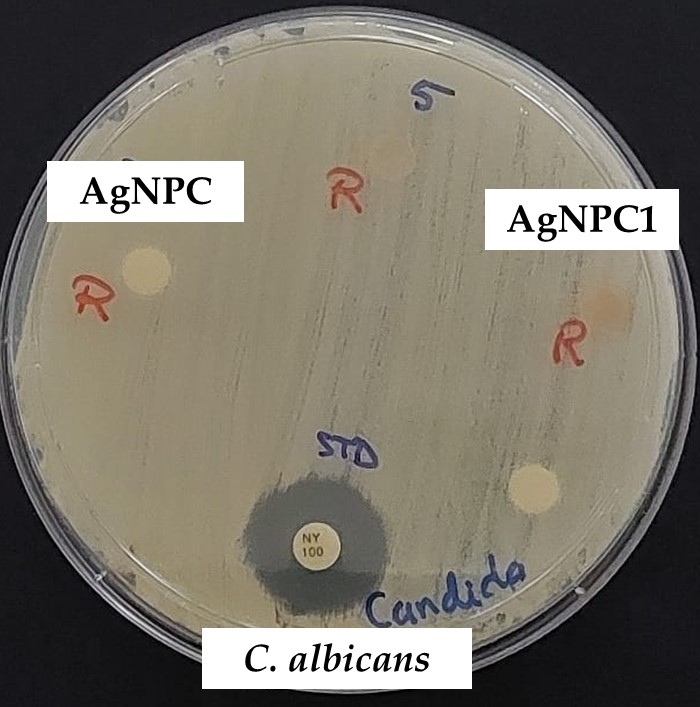

Supplement: Supplementary file 12 [file Image6.jpeg]
